# Discovery of a Modified Tetrapolar Sexual Cycle in *Cryptococcus amylolentus* and the Evolution of *MAT* in the *Cryptococcus* Species Complex

**DOI:** 10.1371/journal.pgen.1002528

**Published:** 2012-02-16

**Authors:** Keisha Findley, Sheng Sun, James A. Fraser, Yen-Ping Hsueh, Anna Floyd Averette, Wenjun Li, Fred S. Dietrich, Joseph Heitman

**Affiliations:** 1 Genetics and Molecular Biology Branch, National Human Genome Research Institute, National Institutes of Health, Bethesda, Maryland, United States of America; 2 Department of Molecular Genetics and Microbiology, Duke University Medical Center, Durham, North Carolina, United States of America; 3 School of Molecular and Microbial Sciences, University of Queensland, Brisbane, Australia; 4 Division of Biology, California Institute of Technology, Pasadena, California, United States of America; University of California San Francisco, United States of America

## Abstract

Sexual reproduction in fungi is governed by a specialized genomic region called the mating-type locus (*MAT*). The human fungal pathogenic and basidiomycetous yeast *Cryptococcus neoformans* has evolved a bipolar mating system (**a**, α) in which the *MAT* locus is unusually large (>100 kb) and encodes >20 genes including homeodomain (HD) and pheromone/receptor (P/R) genes. To understand how this unique bipolar mating system evolved, we investigated *MAT* in the closely related species *Tsuchiyaea wingfieldii* and *Cryptococcus amylolentus* and discovered two physically unlinked loci encoding the HD and P/R genes. Interestingly, the HD (B) locus sex-specific region is restricted (∼2 kb) and encodes two linked and divergently oriented homeodomain genes in contrast to the solo HD genes (*SXI1*α, *SXI2* **a**) of *C. neoformans* and *Cryptococcus gattii*. The P/R (A) locus contains the pheromone and pheromone receptor genes but has expanded considerably compared to other outgroup species (*Cryptococcus heveanensis*) and is linked to many of the genes also found in the *MAT* locus of the pathogenic *Cryptococcus* species. Our discovery of a heterothallic sexual cycle for *C. amylolentus* allowed us to establish the biological roles of the sex-determining regions. Matings between two strains of opposite mating-types (A1B1×A2B2) produced dikaryotic hyphae with fused clamp connections, basidia, and basidiospores. Genotyping progeny using markers linked and unlinked to *MAT* revealed that meiosis and uniparental mitochondrial inheritance occur during the sexual cycle of *C. amylolentus*. The sexual cycle is tetrapolar and produces fertile progeny of four mating-types (A1B1, A1B2, A2B1, and A2B2), but a high proportion of progeny are infertile, and fertility is biased towards one parental mating-type (A1B1). Our studies reveal insights into the plasticity and transitions in both mechanisms of sex determination (bipolar versus tetrapolar) and sexual reproduction (outcrossing versus inbreeding) with implications for similar evolutionary transitions and processes in fungi, plants, and animals.

## Author Summary

Fungal gene clusters mediate sex determination, natural product synthesis, and metabolic functions. Eukaryotic organisms share features of gene cluster formation including translocations, inversions, gene conversion, and suppressed recombination. The *C. neoformans*/*C. gattii* mating-type (*MAT*) locus spans a single >100 kb gene cluster encoding >20 genes, many involved in sex. We examined *MAT* gene cluster evolution in model and pathogenic *Cryptococcus* species. *MAT* was characterized from two closely related species, *T. wingfieldii* and *C. amylolentus*, and is organized into two unlinked gene clusters on different chromosomes. *MAT* organization in these species provides insight into evolutionary transitions from tetrapolar to bipolar mating systems involving fusion of physically unlinked sex-determinants into one contiguous region. These sex determination transitions occurred concomitantly with the origin of the pathogenic species complex from the last common ancestor shared with tetrapolar non-pathogenic species. We discovered a tetrapolar sexual cycle in *C. amylolentus* that generates recombinant meiotic progeny, many of which are infertile. Fertile progeny are biased towards one parental mating-type (A1B1) and may be an evolutionary precursor to unisexual mating of the closely related pathogenic species. This study reveals factors orchestrating gene cluster formation and sex chromosome evolution in fungi, including features shared with animals and plants.

## Introduction

Sexual reproduction is ubiquitous throughout nature, generates population diversity, and has been described extensively in plants, animals, and microorganisms [1]. Sex is both costly and advantageous, and the ubiquity of sexual reproduction suggests that in general its benefits outweigh its costs [2]. In sexually reproducing populations, outbreeding is common, but inbreeding forms of sex also occur that promote clonality. Additionally, unisexual reproduction may be an adaptive virulence strategy for several microbial pathogens [3].

Fungi occur in two mating configurations: bipolar and tetrapolar [4]. In bipolar species, transcription factors that establish mating-type (*MAT*) are encoded by a single locus; in some examples genes encoding pheromones and their receptors are also present [4]. For mating to occur compatible cells must differ at *MAT* (**a** and α), although there are examples of bipolar fungi that also undergo same-sex mating (e.g. *Candida albicans* and *Cryptococcus neoformans* [5]). In tetrapolar species, two physically unlinked genomic regions (i.e. *MAT* loci A and B) control and establish cell identity. These loci are often multiallelic, and alleles must differ at both loci for sexual reproduction to occur. Bipolar mating systems support more efficient inbreeding (50%) and also outbreeding (50%), while tetrapolar systems promote more efficient outbreeding (>99%) and restrict inbreeding (25%) [6]. Ascomycetous yeasts such as *Saccharomyces cerevisiae* and *Candida albicans* are bipolar while basidiomycetous yeasts like *Tremella mesenterica* and *Ustilago maydis* are typically tetrapolar [7]. In contrast to most basidiomycetous species, *Ustilago hordei*, *Coprinellus disseminatus*, *C. neoformans*, and *Cryptococcus gattii* have bipolar mating systems [8], [9], [10], [11], [12].

*C. neoformans* is a haploid, dimorphic fungus that has a bipolar mating system, represented by two alleles, α and **a** [10]. *MAT* spans 100 to 120 kb, and encodes more than 20 genes, many of which are involved in mating. Comparison of the *MAT* gene cluster among the members of the pathogenic *Cryptococcus* species complex revealed that extensive rearrangements and gene conversions have occurred over time even though recombination in this gene cluster is generally suppressed [13], [14], [15], [16]. The sexual cycle and the structure of *MAT* in the pathogenic *Cryptococcus* species have been extensively examined and are well defined [4], [15]. In a laboratory setting, *Cryptococcus* reproduces via either opposite-sex or unisexual reproduction [5], [11], [12], [14], [17], [18]. Mating (α-**a**) initiates with cell-cell fusion, followed by production of a filamentous dikaryon with fused clamp cell connections, and culminates in nuclear fusion and meiosis in the basidia [4], [19]. Meiosis produces four haploid nuclei that undergo mitotic division to produce four chains of basidiospores that germinate into fertile yeasts that can mate with a partner/parent of the opposite mating-type. The major differences in α-α unisexual reproduction is that a monokaryon (instead of a dikaryon) forms, mating can involve two genetically distinct isolates (α_1_-α_2_) or two genetically identical genomes (α_1_-α_1_), and the resulting meiotic spore products are all α.

Fraser et al. proposed that the ancestral form of *MAT* to the pathogenic *Cryptococcus* species was tetrapolar, with the homeodomain (HD) and pheromone/receptor (P/R) genes present in two unlinked sex-determining regions [16]. Sequential rounds of gene acquisition led to the expansion of the ancestral tetrapolar *MAT* loci. In this model, a chromosomal translocation event then fused the unlinked loci into a contiguous region resulting in the formation of a transient tripolar intermediate in which *MAT* is linked in one partner yet unlinked in the other. This unstable intermediate underwent gene conversion to link the other *MAT* locus alleles, one or the other homeodomain gene was lost, and *MAT* was subjected to multiple inversions and gene conversions events to yield the extant bipolar *MAT* locus of *Cryptococcus* 16,20.

The pathogenic *Cryptococcus* species form a monophyletic cluster composed of at least two but possibly as many as six species: *C. neoformans* var. *neoformans*, *C. neoformans* var. *grubii*, and the sibling species *C. gattii* (VGI, VGII, VGIII, VGIV) that all have the potential to infect humans and other animals [21]. A recent multi-locus sequence typing (MLST) phylogenetic study resolved the species relationships in this complex [22]. The monophyletic *sensu stricto Filobasidiella* clade is comprised of the pathogenic species and three closely related saprobic species: *Tsuchiyaea wingfieldii*, *Cryptococcus amylolentus*, and *Filobasidiella depauperata* [22], [23]. The more distantly related *sensu lato* sister clade *Kwoniella* encompasses several saprobic and one aquatic-associated species: *Bullera dendrophila*, *Cryptococcus heveanensis*, *Cryptococcus bestiolae*, *Cryptococcus dejecticola*, and *Kwoniella mangroviensis* [22].

Of these species that are phylogenetically closely related to the pathogenic *Cryptococcus* species complex, sex has recently been described for *C. heveanensis* and *K. mangroviensis* [24], [25]. Specifically, a heterothallic sexual cycle was observed in these two members of the *Kwoniella* clade and basidiospores associated with cruciate-septated basidia are produced during mating. Additionally in *F. depauperata* and *T. mesenterica*, the nature of sex has also been revealed in previous studies and exemplifies homothallic and heterothallic sexual cycles, respectively [12], [26], [27], [28]. The mating structures of *F. depauperata* resemble the basidia and basidiospores of *C. neoformans* and *C. gattii* while *T. mesenterica* mating products are similar to *C. heveanensis* and *K. mangroviensis* [12], [26], [27], [28], [29]. However, no sexual reproduction had been observed in either *C. amylolentus* or *T. wingfieldii*.

A recent study of *C. heveanensis* revealed it has a tetrapolar mating system, i.e. its sexual reproduction is governed by *MAT* comprised of two physically unlinked gene clusters: a multiallelic HD locus (B locus) and a P/R locus (A locus) that is at least biallelic [24]. However, it still remains unclear when the bipolar mating system in *Cryptococcus* pathogenic species first appeared, that is, did it emerge earlier in the common ancestor of the *sensu stricto* group when it split from the *sensu lato* group, or did it evolve later and only in the *Cryptococcus* pathogenic species? Given the close relationship of *T. wingfieldii* and *C. amylolentus* to the pathogenic *Cryptococcus* species complex, understanding their life cycles, as well as their *MAT* loci configurations can provide key insights into the evolution of *MAT* and sexual reproduction in *C. neoformans* and *C. gattii*.

In this study, we provide a detailed description of the heterothallic sexual cycle of *C. amylolentus* that we observed under laboratory conditions. Additionally, we characterized the *MAT* loci of *T. wingfieldii* and *C. amylolentus*, and discovered in both species two physically unlinked gene clusters, one encoding the HD locus and the other encoding the P/R locus. Genes within these clusters include many homologs of *Cryptococcus MAT*-associated genes. Furthermore, our mating assay and genetic analyses of *C. amylolentus* meiotic progeny showed that many meiotic progeny are sterile and one parental type is overrepresented in the meiotic products, suggesting its tetrapolar mating system deviates from the classic model. We discuss the implications of our findings in the context of the evolution of the mating type locus as well as of bipolar sexuality in the *Cryptococcus* species complex. Our findings also provide insights into similar evolutionary processes that drive the formation and function of sex chromosomes in algae, fish, insects, and mammals [14].

## Results

### Characterizing *MAT* in *T. wingfieldii* and *C. amylolentus*

**Figure 1:**
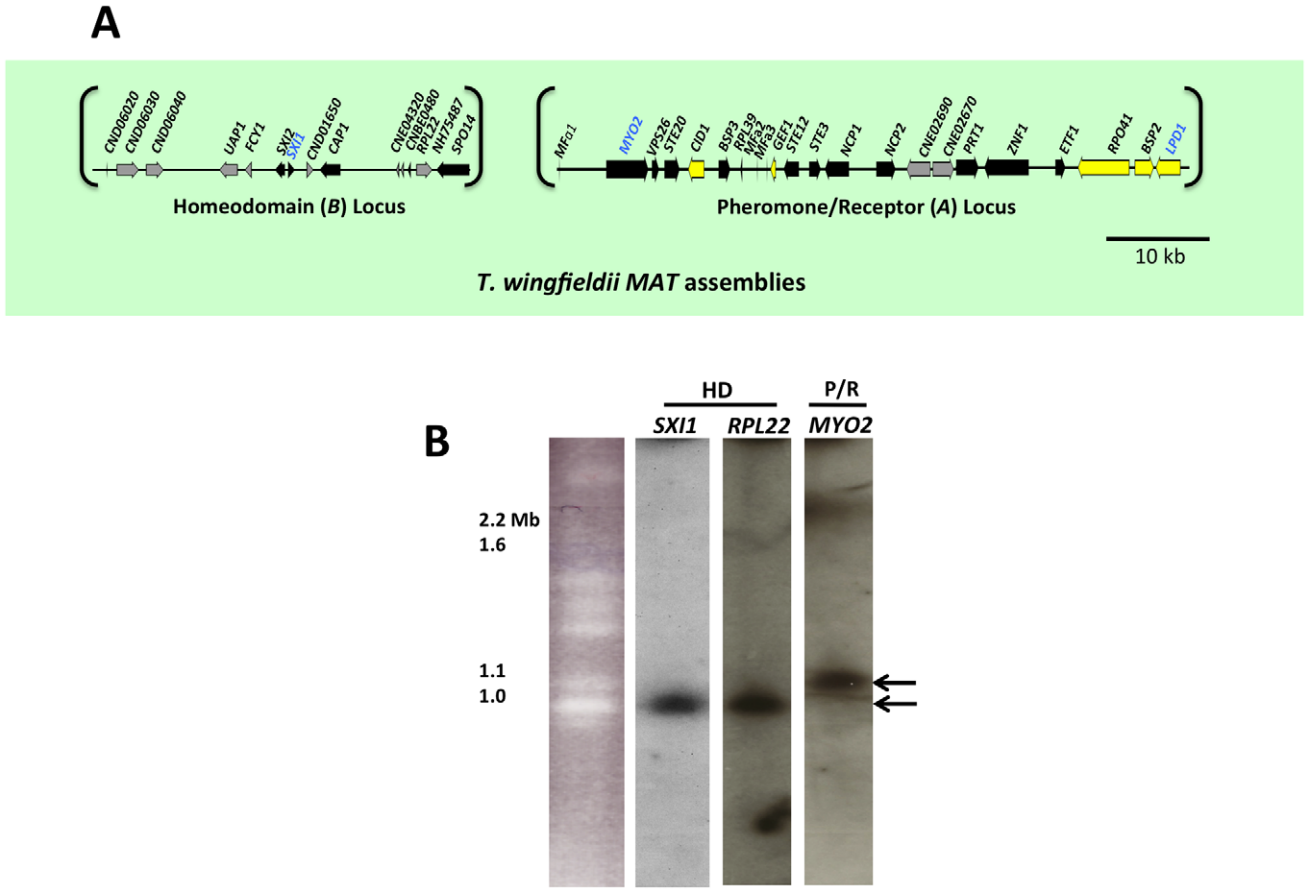
*T. wingfieldii MAT* loci and chromosomal locations. (A) Six fosmids were analyzed to generate the assembly for *T. wingfieldii*. The *MAT* gene probes used to probe the *T. wingfieldii* library are indicated in blue. The HD (*B*) and P/R (*A*) loci are embedded within assemblies that span 40 and 70 kb respectively. Grey arrows indicate genes that either flank *MAT* or are hypothetical genes, black arrows are *Cryptococcus MAT*-specific genes, and yellow indicates the genes most recently acquired into the *Cryptococcus MAT* locus. Scale bar = 10 kb. (B) Chromosomes from *T. wingfieldii* were separated using PFGE, followed by Southern hybridization using three *MAT*-specific probes, two from the HD locus and one from the P/R locus. Arrows depict hybridization of HD genes to an ∼1 Mb chromosome distinct from hybridization of the P/R genes to an ∼1.1 Mb chromosome.

To determine the structure of *MAT* in *T. wingfieldii*, fosmid libraries were constructed from the type strain CBS7118 and probed with several genes within (*MYO2*, *LPD1*, and *SXI1*) or flanking (*FAO1* and *NOG2*) the *C. neoformans MAT* locus. Positive clones (3F11-3A15-5J15 (P/R locus), 2B23-2K10 (HD locus), and 4E07 (*FAO1*), see Figure S1) were pooled and sequenced, resulting in the identification of two candidate *MAT* loci. The *FAO1* gene lies on a distinct fosmid and appears to be unlinked or distant from *MAT*. The region obtained containing the P/R locus spans ∼70 kb and the region obtained containing the HD locus spans ∼40 kb (Figure 1A).

**Figure 2:**
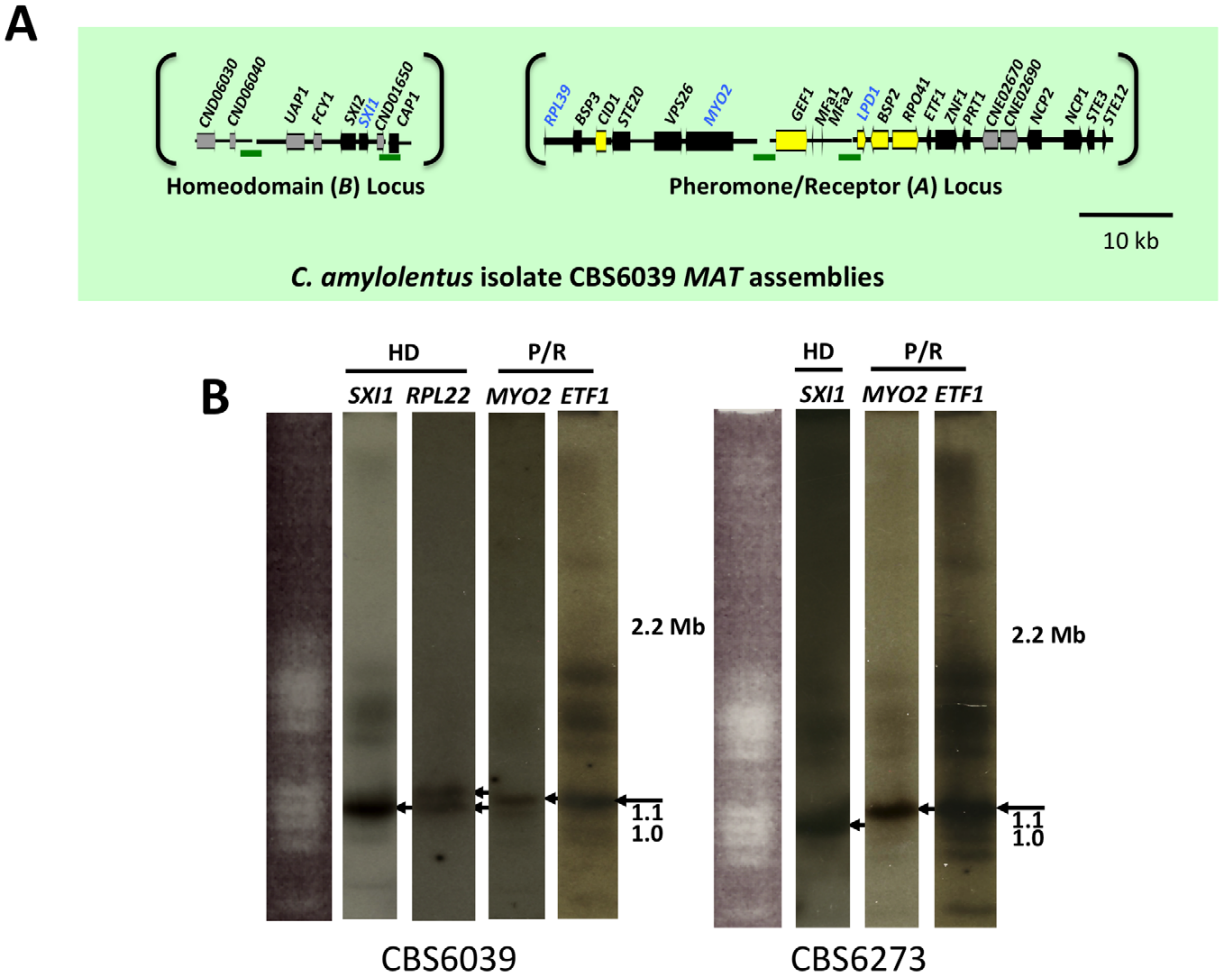
*C. amylolentus MAT* loci and chromosomal locations. (A) Four fosmids were analyzed to generate the assembly for *C. amylolentus*. The *MAT* gene probes used to probe the *C. amylolentus* library are indicated in blue. The HD (*B*) and P/R (*A*) loci are embedded in regions that span 20 and 60 kb respectively. Grey arrows indicate genes that either flank *MAT* or are hypothetical genes, black arrows are *Cryptococcus MAT*-specific genes, and yellow indicates the genes more recently acquired into the *Cryptococcus MAT* locus. Several gaps remain in the *MAT* loci of *C. amylolentus*. Scale bar = 10 kb. Green bars under the assembly denote gaps in sequence. (B) Chromosomes from *C. amylolentus* were separated using PFGE, and analyzed by Southern hybridization using three *MAT*-specific probes, one from the HD locus and two from the P/R locus. The *RPL22* gene was also used as a probe. Arrows depict hybridization of the HD and P/R locus probes to distinct chromosomes (∼1.1 and ∼1.15 Mb).

**Figure 3:**
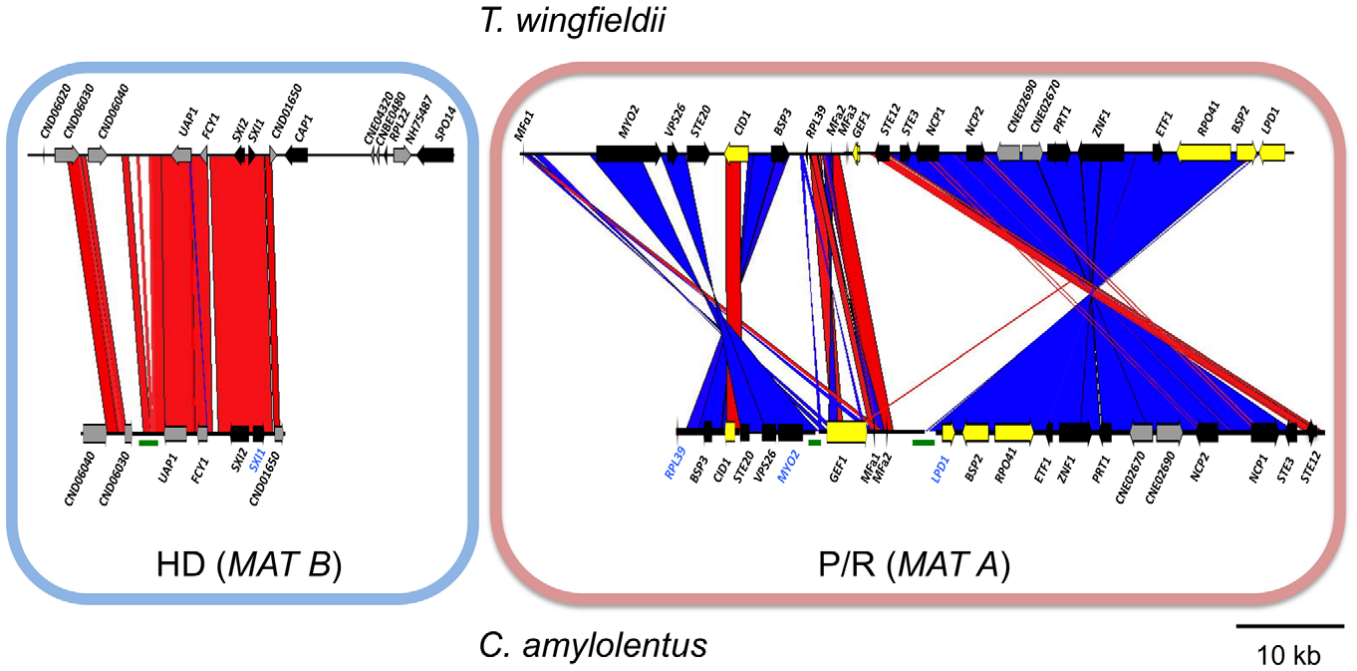
Synteny analysis of *MAT* sequences from *T. wingfieldii* and *C. amylolentus*. On the left is the comparison of the HD (*MAT B*) locus, while the comparison of the P/R (*MAT A*) locus is shown on the right. Red lines connecting *T. wingfieldii* and *C. amylolentus* sequences denote conserved gene order; while blue lines indicate inverted orientations of the sequences from the two species. Green bars under the assembly denote sequence gaps in the assembled contigs.

We also cloned and sequenced *MAT* in the saprobic yeast-like, sibling species *C. amylolentus* (type strain CBS6039) employing the same approach. Fosmid libraries were generated and probed with *MYO2*, *LPD1*, *RPL39*, and *SXI1*. Primers specific for *MAT* genes in *T. wingfieldii* were used to generate probes for *C. amylolentus* and the identity of each probe was confirmed via cloning and sequencing. Positive clones (4E01 (*SXI1*), 4E22 (*MYO2*), and 3H19 (*LPD1*), see Figure S2) were individually sequenced and assembled into two *MAT* loci. An additional fosmid (3N14 (*RPL39*), see Figure S2) was later identified and sequenced via primer walking. The regions that were sequenced span ∼20 kb and ∼60 kb respectively, and each contain two small sequence gaps (Figure 2A and Figures S3 and S4). The linear order of the fragments in the P/R locus was determined based on Southern blotting. Specifically, genomic DNA from CBS6039 and CBS6273 was digested with five restriction enzymes (BamHI, BglI, ClaI, EcoRI, and NcoI) and Southern blot analysis was performed with probes hybridizing to the ends of each contig in the P/R assembly of *C. amylolentus*. The gene content in *MAT* appears to be largely conserved between *T. wingfieldii* and *C. amylolentus*. However, our Southern blot analysis indicated at least two major inversions exist between the P/R regions of these two species (Figure 3).

**Figure 4:**
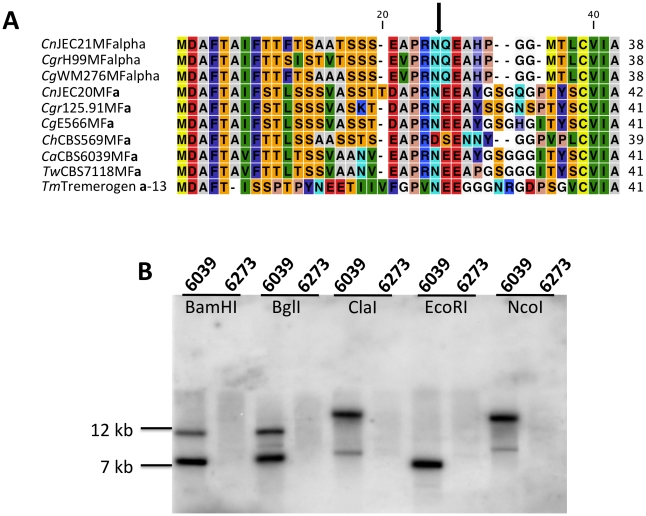
Analysis of the pheromone/receptor genes in *C. amylolentus*. (A) Sequence alignments of the pheromone gene in *C. neoformans* var. *neoformans* JEC21 *MFα*, *C. neoformans* var. *grubii* H99 *MFα*, *C. gattii* WM276 *MFα*, *C. neoformans* var. *neoformans* JEC20 *MF* **a**, *C. neoformans* var. *grubii* 125.91 *MF* **a**, *C. gattii* E566 *MF* **a**, *C. heveanensis* CBS569 *MF* **a**, *C. amylolentus* CBS6039 *MF* **a**1, *T. wingfieldii* CBS7118 *MF* **a**1, and *T. mesenterica* ATCC24925 Tremerogen a-13. The black arrow denotes the predicted cleavage site. The pheromone receptor gene, *STE3*, is *MAT* specific. (B) Genomic DNA from the two *C. amylolentus* strains was digested with BamHI, BglI, ClaI, EcoRI, or NcoI and Southern blot analysis was performed using the *STE3* PCR product from CBS6039 as a probe. For each enzyme digestion, CBS6039 was in the left lane and CBS6273 was in the right lane.

**Figure 5:**
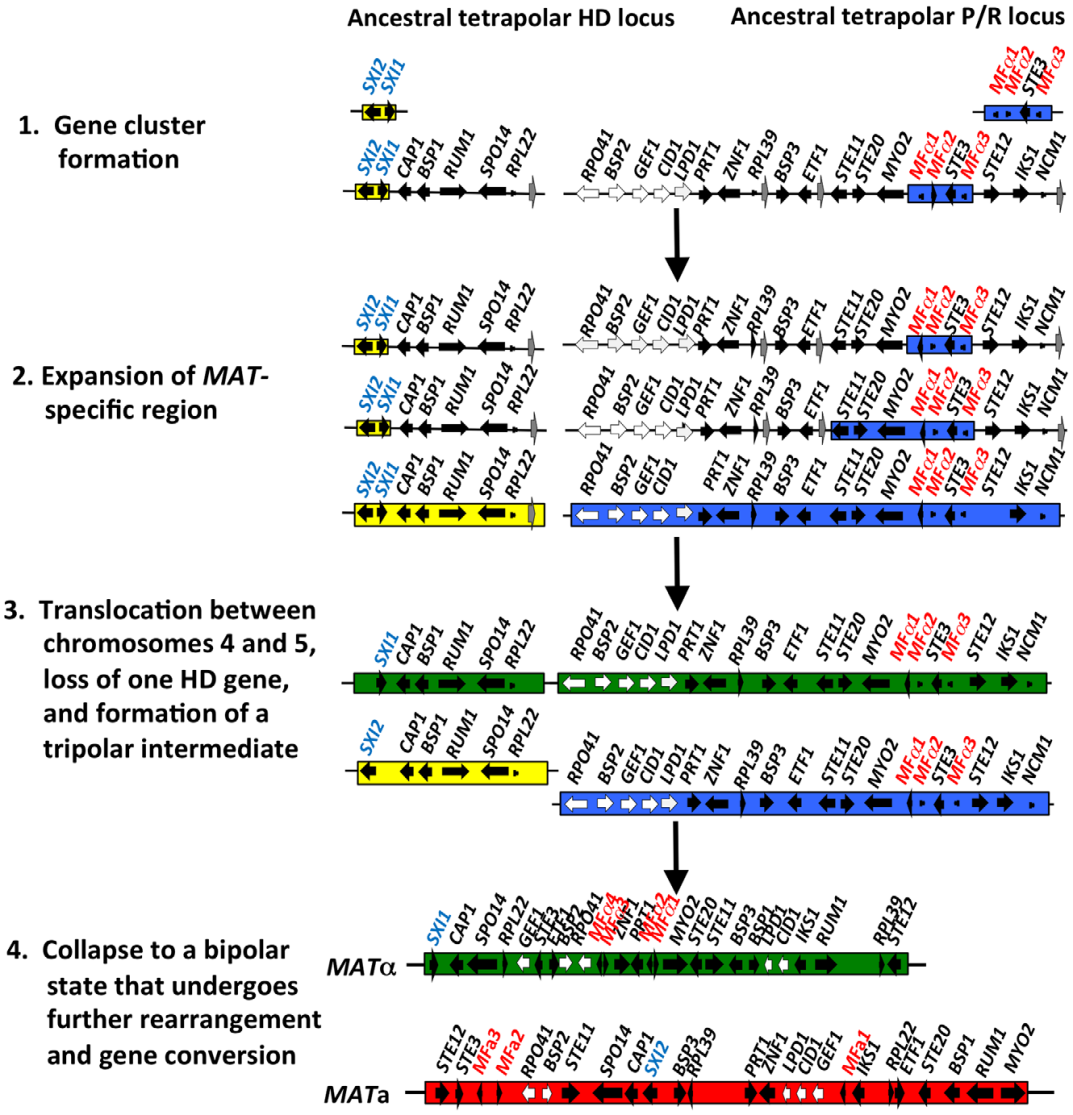
Model for the evolution of the mating-type locus in the pathogenic *Cryptococcus* species. The physically unlinked ancestral tetrapolar HD and P/R loci contained both homeodomain genes and the pheromone/receptor genes respectively. Additional genes were acquired into both loci, expanding the *MAT*-specific region. A translocation event occurred, likely between chromosomes 4 and 5 of *Cryptococcus*, resulting in the formation of a transient tripolar intermediate and one of the HD genes was lost. The hypothetical genes (grey arrows) relocated, likely through a translocation event, to the telomeric ends of chromosome 4. The unstable tripolar intermediate later collapsed to a bipolar state. The fused loci were subjected to further gene rearrangement and gene conversion events, which led to the formation of the bipolar alleles of the pathogenic *Cryptococcus* species. White arrows indicate the five genes most recently acquired into *Cryptococcus MAT* locus and black arrows are *MAT*-specific genes present in the pathogenic *Cryptococcus* species.

Analysis of the *MAT* sequences obtained from *T. wingfieldii* and *C. amylolentus* revealed that the gene content of these regions are similar to the *C. neoformans* and *C. gattii MAT* alleles [10], [16]. In both sibling species, orthologs of both *SXI1* and *SXI2* are present in the HD locus implicating this as the ancestral configuration (Figure 1 and Figure 2). The orientation of the homeodomain transcription factors mirrors the organization of the paired, divergently transcribed genes, *bE* and *bW*, in the tetrapolar basidiomycete *U. maydis* [29], [30]. In contrast, in *C. neoformans* and *C. gattii*, only one HD gene is present and *SXI1*α is specific to the α allele while *SXI2* **a** is specific to the **a** allele. The region corresponding to the P/R locus contains the mating pheromone genes, the pheromone receptor gene *STE3*, and the five genes that were hypothesized to be those most recently acquired by the *Cryptococcus MAT* locus (*LPD1*, *RPO41*, *BSP2*, *CID1*, and *GEF1*). In *T. wingfieldii*, three pheromone genes (*MF* **a** *1* and *MF* **a** *3* are identical while *MF* **a** *2* differs in only one amino acid) are present and share greater identity with the *MF* **a** genes of *C. gattii* with an identity of 80% compared to 70–75% shared with the *MFα* pheromone gene (Figure 4A). The P/R region in *C. amylolentus* differs from *C. heveanensis* in that the pheromone genes are located >30 kb away from *STE3* whereas in *C. heveanensis* these genes are closely linked [24]. Moreover *LPD1*, *STE11*, *ZNF1*, and *IKS1* are not within the P/R locus of *C. heveanensis* [24], while the P/R region is more extensive in *C. amylolentus* and spans >60 kb (Figure 2 and Figure S2). In *C. amylolentus*, two pheromone genes (*MF* **a** *1* and *MF* **a** *2* differ in only two amino acids) have been identified and share 73% identity with the MF**a** protein product of *C. neoformans* and 65–70% identity with the *MFα* pheromone gene. In summary, both *SXI1* and *SXI2* were present in the ancestral HD locus of the *sensu stricto Filobasidiella* clade. Thus, loss of one or the other HD gene occurred during the evolution of *MAT* in the pathogenic *Cryptococcus* species. Additionally, the five genes most recently acquired by the *Cryptococcus MAT* locus are linked to the ancestral P/R locus and thus appear to have been acquired into the expanding *MAT* A locus rather than entrapped by the *MAT* fusion event, in contrast to an earlier evolutionary model, suggesting a revision to the model (Figure 5) [16].

In *T. wingfieldii* and *C. amylolentus*, the *FCY1* and *UAP1* genes flank the 5′ end of the *MAT* HD locus, similar to *C. neoformans*/*C. gattii*, but *FAO1* is unlinked and present elsewhere in the genome. We observed that *STE11* is not present in the P/R locus but, based on PCR analysis, it is located elsewhere in the genome in both *T. wingfieldii* and *C. amylolentus* (data not shown). In the *MAT* locus of the pathogenic *Cryptococcus* species, *STE11* is present. In *C. heveanensis*, *STE11* is linked to but distant from the P/R locus and this may represent the ancestral configuration with retention in *C. neoformans* and *C. gattii* and translocation out of *MAT* in *C. amylolentus* and *T. wingfieldii* [24]. In *T. wingfieldii*, the flanking gene at the 3′ end of *MAT*, *NOG2*, was used as a probe. It was present in a single contig within the larger fosmid assembly of *T. wingfieldii*, but has not been linked to either the HD or P/R loci contigs. PCR analysis (using gap closure) revealed that *LPD1* is linked to the P/R locus, although this gap remains to be sequenced. Interestingly, *NCP1* and *NCP2* are duplicated genes in *T. wingfieldii* and *C. amylolentus* but not in the pathogenic *Cryptococcus* species. The *NCP1/2* genes are also duplicated in *C. heveanensis* [24], suggesting this configuration might be ancestral.

We also identified several hypothetical genes (CND06020, CND06030, CND06040, CND01650, CNBE0480, CNE02690, and CNE02670) with *C. neoformans* genes as the most closely related homolog in other sequenced fungal genomes. Four of these genes reside on chromosome 4 and two on chromosome 5 of *C. neoformans*, indicating that translocation (intra- and inter-chromosomal) events may have occurred between these two chromosomes during the evolution of *MAT* in the pathogenic *Cryptococcus* species [16], [28]. In *C. heveanensis*, *F. depauperata*, and *T. mesenterica* there is additional evidence for similar exchanges between chromosomes [14], [24], [28].

A considerable level of synteny exists across both *MAT* loci in *T. wingfieldii* and *C. amylolentus*, but we also observed at least two major inversion events that have occurred between the two genomes (highlighted in blue in the P/R locus, Figure 3). Comparison of each sibling species to the *C. neoformans* serotype D strain JEC21 revealed extensive gene rearrangements and inversions present throughout *MAT* (Figure S5), similar to the comparisons of *MAT* within the *C. neoformans/C. gattii* species complex. The arrangement of the *MAT* loci in *T. wingfieldii* and *C. amylolentus* corresponds to an evolutionary intermediate in *MAT* evolution in which the loci (or their linked gene repertoire) have expanded but not yet fused.

### The HD and P/R loci are physically unlinked in *T. wingfieldii* and *C. amylolentus*

Analysis using pulsed-field gel electrophoresis and Southern hybridization demonstrated that the HD and P/R loci are physically unlinked in *T. wingfieldii*, as well as in both strains of *C. amylolentus* (CBS6039 and CBS6273). Each genome has approximately 10–12 chromosomes ranging in size from 800 kb to 2.2 Mb. Three genes were used to probe the *T. wingfieldii* chromosomes, two from the HD locus, *SXI1* and *RPL22*, and one from the P/R locus, *MYO2* (Figure 1B). For *C. amylolentus*, a total of three genes were used as probes: one from the HD locus, *SXI1*, and two from the P/R locus, *MYO2* and *ETF1* (Figure 2B). From the chromoblot analysis, the two loci are located on separate chromosomes (∼1.1 and 1.15 Mb) in both of the sibling species. That the HD and P/R loci are located on different chromosomes suggests a tetrapolar mating configuration for both *sensu stricto* species *T. wingfieldii* and *C. amylolentus*. Moreover, given the finding that other more distant outgroup species (*C. heveanensis*, *T. mesenterica*) are also tetrapolar [24], the most parsimonious interpretation is that the tetrapolar configuration represents the ancestral form of *MAT* and the bipolar state observed for the pathogenic *Cryptococcus* species therefore arose even more recently than revealed by previous studies of the more distantly related *sensu lato* species *C. heveanensis* [24]. Thus, the organization of *MAT* in the sibling species resembles key aspects of the proposed intermediates in the evolution of bipolar *MAT* in the pathogenic *Cryptococcus* species from a tetrapolar ancestor.

### Identification of key genes that define *MAT*

*MAT* is defined as a gene cluster (containing either HD and/or P/R genes) whose sequence is divergent between two strains of opposite mating-types. Based on the characterized structure of *MAT* in both species, we sought to determine which genes in each region govern and control sexual identity. The lack of additional *T. wingfieldii* strains has made it difficult to assess experimentally whether it has a sexual cycle and, if so, which genes are involved. Fortunately, in *C. amylolentus* two strains are available and this enabled our analysis of *MAT* and sex in this species resulting in the discovery of an extant sexual cycle (described below).

**Figure 6:**
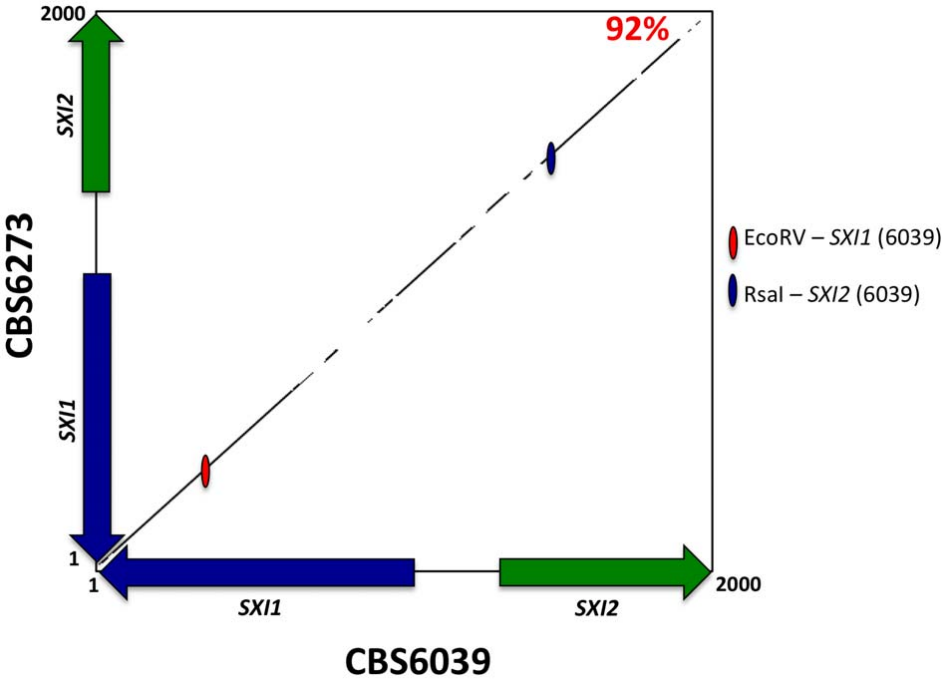
The homeodomain genes, *SXI1* and *SXI2*, define *MAT*. A percent identity plot of both *C. amylolentus* strains, CBS6039 and CBS6273, comparing the *SXI1* and *SXI2* dimorphic region in the HD locus. The red ellipsoid represents an EcoRV site, which only cleaves *SXI1* in CBS6039 while the blue ellipsoid represents an RsaI site, which only cleaves *SXI2* in CBS6039.

Regions that define *MAT* typically display polymorphisms when comparing sequences from strains of opposite mating-type while the genes that flank *MAT* share a much higher level of identity (≥99%). The *SXI1* and *SXI2* dimorphic region defines the diverged region of the *MAT B* HD locus in *C. amylolentus*. We aligned the nucleotide sequences and performed a matrix comparison for the dimorphic region (∼2 kb) spanning the *SXI1* and *SXI2* genes in CBS6039 and CBS6273. The diversity lies in the region between the two genes, and their divergently oriented 5′ regions span roughly 600 bp with a similarity score of 92% (Figure 6). This region encodes the N-terminal dimerization regions known to be variable and which also defines alleles in other species (please see Text S1, and Figures S10, S11, S12, S13 for further information on analyses of HD dimorphic region in meiotic progeny). Moreover, the sequence length for CBS6273 is slightly shorter than for CBS6039 at the 3′ end of the region we sequenced for the *SXI2* gene. In summary, the *SXI1* and *SXI2* genes span ∼2 kb and define the *B MAT* locus in *C. amylolentus*. Although it is not yet clear whether there are any other sexually dimorphic regions beyond *SXI1* and *SXI2* (which could reflect expansion of the HD locus), our analysis based on PCR assay showed that the areas flanking the *SXI1* and *SXI2* genes are conserved enough between CBS6039 and CBS6273 that primers designed based on CBS6039 sequence amplify corresponding regions from CBS6273 (data not shown).

To determine whether the pheromone receptor gene *STE3* lies within the *A* P/R mating-type locus, we performed Southern blot analysis using genomic DNA from the two strains of *C. amylolentus*. The *STE3* PCR product derived from CBS6039 was used as a probe, and only hybridized to the lanes containing CBS6039 DNA with no hybridization to CBS6273 (Figure 4B). This analysis provides evidence that the *STE3* gene differs between the two *C. amylolentus* strains and the pheromone receptor gene is also linked to mating-type. Extensive additional Southern and PCR data (summarized in Figures S2, S3, S4) document that the sequence divergent region of the P/R locus spans more than 60 kb encompassing multiple genes (mating pheromone genes, *STE3*, *STE12*, and *STE20* among others). This contrasts with *C. heveanensis* in which the P/R locus is more restricted, *STE3* and the *MF* pheromone genes are closely linked, and the *LPD1*, *STE11*, *ZNF1*, *MYO2*, and *IKS1* genes are linked to but not within *MAT* [24]. In conclusion, in *C. amylolentus* a tetrapolar mating system with physically unlinked HD and P/R loci appears to define mating-type identity, and the P/R locus has expanded considerably compared to *C. heveanensis*, revealing an evolutionary intermediate in the transition from the tetrapolar to bipolar state that is even more closely related to the pathogenic species complex.

### Phylogenetic analysis of *MAT* related genes in *C. amylolentus* and *T. wingfieldii*

**Figure 7:**
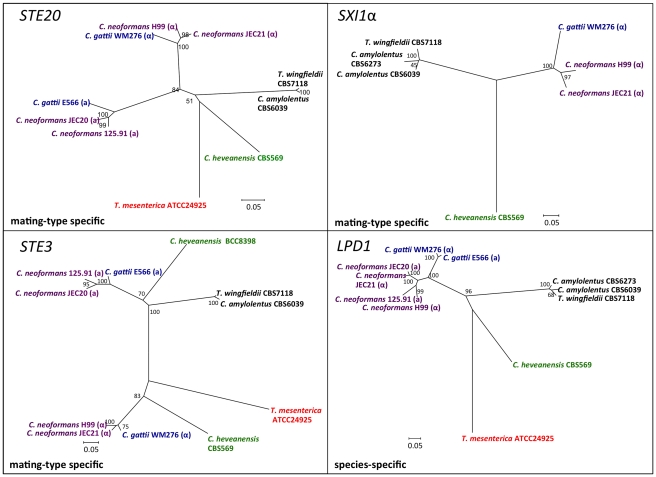
Phylogenetic patterns of four *C. amylolentus MAT* genes. The phylogenetic relationships of *C. amylolentus* to the pathogenic *Cryptococcus* species and neighboring taxa based on four genes, *GEF1*, *CID1*, *SXI1*, and *SXI2*, are shown. *GEF1* and *CID1* display a species-specific phylogeny and the *SXI1* and *SXI2* alleles are very diverged from the pathogenic *Cryptococcus* species. The trees were constructed using the Neighbor-Joining method implemented in the software MEGA4. Bootstrap values on tree branches were calculated from 500 replicates. (α) indicates strains with the *MAT*α locus, and (**a**) indicates strains with the *MAT* **a** locus.

We conducted phylogenetic analysis of several genes that are located within the *MAT* locus of *C. neoformans* (*CID1*, *ETF1*, *GEF1*, *LPD1*, *STE3*, *STE12*, *STE20*, *SXI1*, and *SXI2*). This analysis included *C. neoformans* var. *neoformans*, *C. neoformans* var. *grubii*, and *C. gattii* representatives from the pathogenic species cluster [10] and the closely related sibling species *C. amylolentus* and *T. wingfieldii*, as well as the outgroup species *C. heveanensis* and *T. mesenterica* [24]. Based on the phylogeny of the species within the *C. neoformans* pathogenic species cluster, these genes can be classified into three different groups: species specific (*CID1*, *GEF1*, *LPD1*), mating-type specific (*ETF1*, *STE3*, *STE12*, *STE20*), and mating-type unique genes (*SXI1*, *SXI2*) (Figure 7 and Figures S6 and S7).

The species- specific phylogeny of *CID1*, *GEF1*, and *LPD1* is consistent with the hypothesis that this region has been recruited into the *MAT* locus of *C. neoformans* during the transition from a tetrapolar to a bipolar mating system. For the sex-unique genes in the *C. neoformans* species complex, *SXI1* and *SXI2*, *SXI2* showed a considerably higher level of polymorphism between the two alleles from the CBS6039 and CBS6273 *C. amylolentus* isolates (Figure S7). *ETF1* might have gained its mating-type specific divergence in the *C. neoformans* species complex after the common ancestor of the species complex split from the other sibling species while *STE3*, *STE12*, and *STE20* all have mating type specific phylogenetic patterns within the *C. neoformans* species complex. In *C. amylolentus*, PCR primers designed based on CBS6039 sequences were only able to amplify these genes from CBS6039, but not from CBS6273, indicating the existence of considerable polymorphisms between the two alleles of CBS6039 and CBS6273 for each of these three genes. This is consistent with the mating type specific pattern observed within the *C. neoformans* species complex. Additionally, for *STE3* and *STE12*, the clusters of *C. amylolentus* and *T. wingfieldii* are more closely related to the *MAT* **a** alleles of *C. neoformans* species complex, suggesting a possible common origin of these alleles, as well as an early involvement of the *STE3* and *STE12* genes in the evolution of mating type determination.

### Discovery of the sexual cycle in *C. amylolentus*

**Figure 8:**
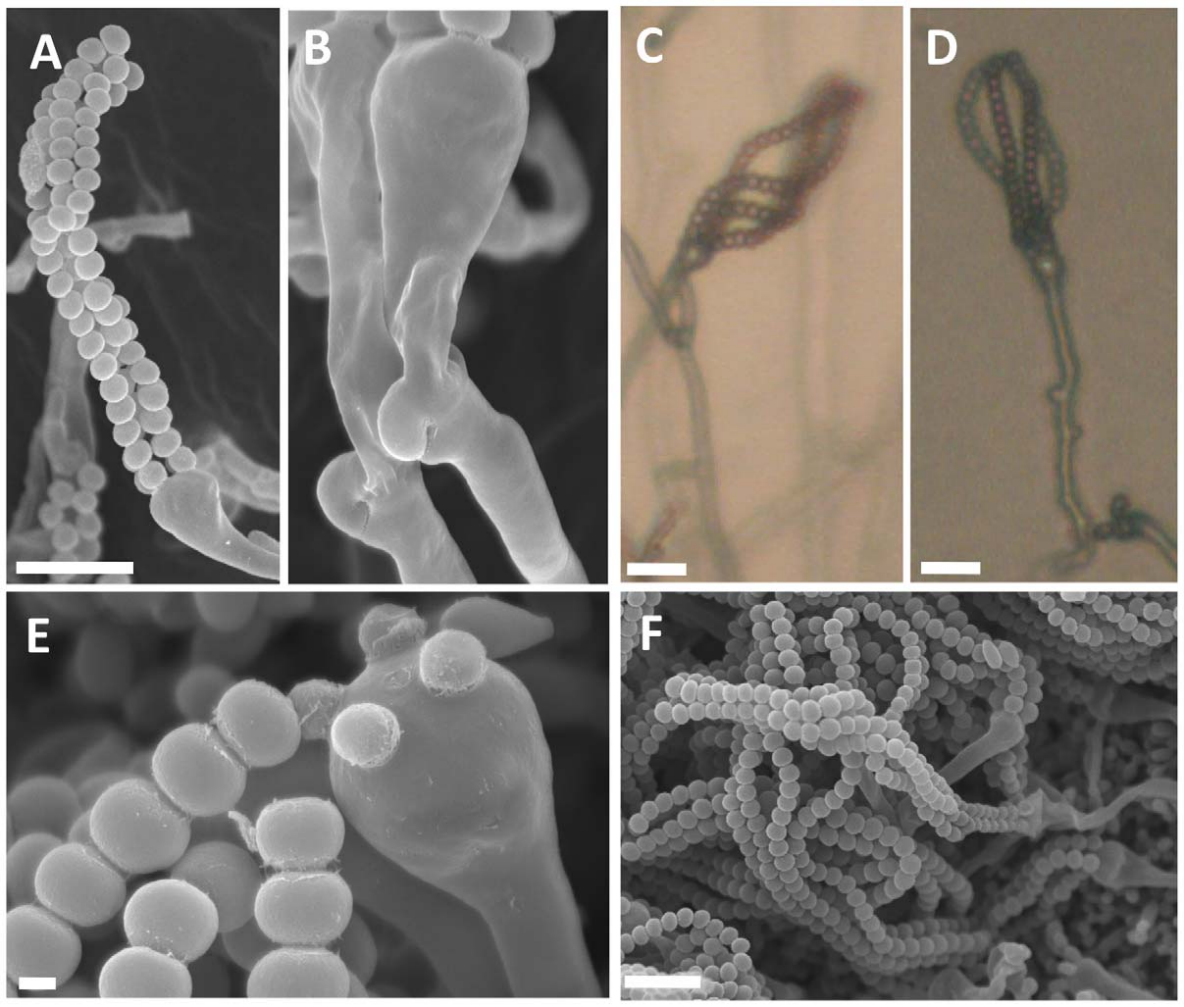
Sexual reproduction of *C. amylolentus*. Microscopic examination of mating structures produced during sex between the two *C. amylolentus* strains, CBS6039 and CBS6273, on V8 (pH = 5) medium incubated in the dark at room temperature for 2 weeks. (A) SEM of basidiospores attached to basidia. Scale bar represents 10 µm. (B) SEM of fused and unfused clamp connections. (C and D) Light microscopy at a magnification of 20X of hyphal filaments, basidia, and basidiospores, scale bar = 10 µm. (E) Basidium with youngest spores attached and associated detached spore chains, scale bar = 1 µm. (F) A cluster of basidiospores and basidia. Scale bar = 10 µm.

Following definition of the mating-type locus for both sibling species, we sought to identify a sexual cycle for *C. amylolentus* and *T. wingfieldii* to determine whether the *A*, *B*, or both *A* and *B MAT* loci control sexual reproduction. It was previously thought that both of these sibling species were asexual [31]; however, we discovered an extant heterothallic sexual cycle for *C. amylolentus*. We conducted mating assays and found the following optimal conditions: V8 pH = 5 solid medium with incubation for one week or longer at room temperature in the dark. The cross between *C. amylolentus* strains CBS6039 and CBS6273 produced hyphae with fused clamp connections and aseptated basidia terminating in four long individual spore chains (please see further discussion on strains CBS6039 and CBS6273 in Text S1, and formal description of mating in Materials and Methods section), similar to matings in *C. neoformans* and *C. gattii*. Sterigmata were not observed (Figure 8A–8F). A marked, obvious feature is the shape of the spores which are ellipsoid in the pathogenic species [32] whereas *C. amylolentus* spores are round and similar in size to yeast cells. Crosses of either *C. amylolentus* strain with *T. wingfieldii* were infertile. Because there is only one strain of *T. wingfieldii* available, *T. wingfieldii* might be fertile in the presence of a suitable partner, similar to the two interfertile *C. amylolentus* strains, or it could be a sterile isolate.

In *C. amylolentus*, we observed that the periphery of some mating patches contains a mixture of both monokaryotic hyphae and sectors in which mating occurs to produce dikaryotic hyphae indicative of sexual reproduction. The dikaryotic sectoring phenotype is present in most mating patches and also serves as a visual assay for mating. The structures produced during the sexual cycle of *C. amylolentus* were visualized in greater detail by microscopy. The four spore chains are each very long consisting of >15 (quantified by counting 10 individual basidia) spores per chain and clamp cell connections are visible by light microscopy and SEM (Figure 8A–8F). Based on fluorescence microscopy with Hoechst 33258 or Sytox green, dikaryotic hyphae and both uni- and occasional bi-nucleate spores were observed (Figure S8A–S8D). In the *Filobasidiella* lineage, *C. neoformans* and *C. gattii* produce both dikaryotic (heterothallic) and monokaryotic (homothallic) hyphae while *F. depauperata* produces only monokaryotic hyphae. The presence of dikaryotic hyphae in *C. amylolentus* provides evidence that opposite-sex mating occurs during the sexual cycle [11], [12]. Additionally, the presence of two nuclei in some basidiospores could result from either a mitotic nuclear division in the spore or packaging of two nuclei into some spores (as occurs in pseudo-homothallic species) [33].

Interestingly, the cap of the spore chain represents a quartet of basidiospores. These spores are the oldest in the spore chain and remain tightly attached to each other. Younger spores in the four spore chains remain attached to the preceding and following spores in the chain but often not to their meiotic siblings in the other three spore chains. Thus, the quartet spore cap appears to tether the ends of the spore chains together. This feature has not been described in the pathogenic *Cryptococcus* species. In summary, microscopic examination of mating structures in *C. amylolentus* has revealed both shared hallmarks with sexual reproduction in the pathogenic *Cryptococcus* species and novel features.

### Genotypic analysis of meiotic progeny

To determine if recombination occurs, and to further assess whether the mating system of *C. amylolentus* is tetrapolar or bipolar, we performed microdissection of random progeny (F1 set 1) and individual spore chains (F1 set 2) followed by molecular genotyping analysis for both *MAT* markers and a genome-wide set of RAPD markers. We designate the CBS6039 parent as A1B1 and the CBS6273 parent as A2B2, according to the designation used for a tetrapolar mating system and our findings, assigning A as the P/R locus and B as the HD locus (as in *T. mesenterica*, *C. heveanensis*, and *U. maydis* [24], [26]).

For F1 set 1 (F1S1), a total of 40 spores were dissected and 28 (70%) germinated (Tables S1 and S2). The progeny were all haploid based on FACS analysis with *C. neoformans* as reference (data not shown). Genotyping using *MAT* markers and RAPD markers revealed that most of the progeny inherited all of the parental alleles from CBS6039 (A1B1) (Tables S1 and S2) and did not appear to be meiotic recombinants. Of the 28 progeny, three (11%) did show recombination within the P/R locus (#17, 27, and 28), whereas only one additional progeny (3.5%) exhibited reassortment between the P/R and HD loci (#18). We hypothesize that this is likely due to the dissection of a mixture of yeast cells, blastospores (mitotic pre-meiotic cells produced by budding from the hyphae or clamp cells), and basidiospores (meiotic sexual spores) [5], [34], which are all morphologically similar for this species. Similar to *C. neoformans*, in *C. amylolentus* blastospores can be generated from the clamp cell, and the following repeated mitotic events tend to produce a cluster of cells at the hyphal septa. This may explain why we did not observe an equal distribution of markers from the two parental strains among the blastospores, as they could have been mitotic products from one common parental blastospore. That many isolates in F1S1 could be blastospores is also supported by analysis of the mitochondrial genome segregation (as shown below) that revealed a majority of this progeny set possess nuclear and mitochondrial genomes inherited from different parents. Remarkably, 22 (78%) of the progeny are sterile and unable to undergo sexual reproduction with either parent or their F1 siblings. It is interesting that progeny that appear to be derived from blastospores are, for unknown reasons, frequently sterile.

To analyze meiotic basidiospores specifically, we dissected F1 set 2 (F1S2) from four well-resolved individual spore chains (one chain each from four different basidia). The germination frequency was 91% (31/34), and 58% (18/31) of the progeny were sterile with both parents (Table 1). All of this progeny set were also haploid based on FACS analysis (data not shown). Molecular analysis of this set using the same six *MAT A* or *B* genes revealed that 64.5% (20/31) of the progeny resembled one or the other parent (A1B1 or A2B2) while the other ∼35% exhibit evidence of recombination within the P/R locus and/or between the HD and P/R loci (i.e. A1B2 or A2B1 progeny) (Table 1). In contrast to the first F1 progeny set (F1S1), genotyping of the spore chain derived progeny set (F1S2) using RAPD markers revealed extensive recombination (Table 2). Linkage analyses clustered markers analyzed in this study into several linkage groups, indicating independent inheritance of markers (data not shown). In addition, analysis of the markers implemented in this study revealed that for each marker, the two parental alleles were equally inherited across the entire progeny set (Table 2). Specifically, for each marker, the percentages of the CBS6039 allele ranged between 35% and 71%, which did not show any significant bias toward one parental allele (chi-square test, P>0.05). Similarly, the percentage of the CBS6039 allele that each progeny inherited ranged from 25% to 80%, and again these values reflect equivalent inheritance of alleles from either parent (chi-square test, P>0.05). Moreover, we observed that meiotic recombination in *C. amylolentus* resulted in the generation of new combinations of alleles in the progeny given the multiple genotypes present in the different spore chains analyzed. The observed high level of recombination and equivalent inheritance of the two parental alleles support the conclusion that meiosis occurs in *C. amylolentus*.

**Table 1:** Summary of mating abilities and genotypes at the *MAT* genes of F1 set 2 and F2 progeny.

	Strain	Mating as	B locus^1^		A locus^1^			
			*SXI2*	*SXI1*	*RPL39*	*GEF1*	*ETF1*	*STE3*
Parental strains	CBS6039	A1B1	a	a	a	a	a	a
	CBS6273	A2B2	b	b	b	b	b	b
Basidium A	F1S2-1	sterile	b	b	b	b	b	b
	F1S2-2	sterile	b	b	b	b	b	b
	**F1S2-3**	**A1B1**	**a**	**a**	**a**	**a**	**a**	**a**
	**F1S2-4**	**A1B1+A2B2**	**a**	**a**	**a**	**a**	**a**	**a**
	F1S2-5	sterile	a	a	a	a	a	a
	F1S2-6	sterile	a	a	a	a	a	a
	F1S2-7	sterile	b	b	b	b	b	b
Basidium B	**F1S2-8**	**A1B1**	**a**	**a**	**a**	**a**	**a**	**a**
	F1S2-9	sterile	a	a	b	b	b	b
	***F1S2-10***	***A1B2***	***b***	***b***	***a***	***a***	***a***	***a***
	**F1S2-11**	**A1B1**	**a**	**a**	**a**	**a**	**a**	**a**
	**F1S2-12**	**A1B1**	**a**	**a**	**a**	**a**	**a**	**a**
	**F1S2-13**	**A2B2**	**b**	**b**	**b**	**b**	**b**	**b**
	**F1S2-14**	**A1B1**	**a**	**a**	**a**	**a**	**a**	**a**
	F1S2-15	sterile	b	b	b	a	a	a
	***F1S2-16***	***A1B2***	***b***	***a***	***b***	***a***	***a***	***a***
	F1S2-17	sterile	b	b	b	b	b	b
Basidium C	**F1S2-18**	**A1B1**	**a**	**a**	**a**	**a**	**a**	**a**
	F1S2-19	sterile	b	b	b	b	b	b
	**F1S2-20**	**A1B1**	**a**	**a**	**a**	**a**	**a**	**a**
	F1S2-21	sterile	b	b	b	b	b	b
	**F1S2-22**	**A1B1**	**a**	**a**	**a**	**a**	**a**	**a**
	F1S2-23	sterile	b	b	b	b	b	b
	**F1S2-24**	**A1B1**	**a**	**a**	**a**	**a**	**a**	**a**
Basidium D	F1S2-25	sterile	a	a	b	b	b	b
	F1S2-26	sterile	a	a	b	b	b	b
	F1S2-27	sterile	a	a	b	b	b	b
	F1S2-28	sterile	a	a	b	b	b	b
	F1S2-29	sterile	a	a	b	b	b	b
	F1S2-30	sterile	a	a	b	b	b	b
	F1S2-31	sterile	a	a	b	b	b	b
Basidium E	***F2-1***	***A2B1***	***a***	***a***	***b***	***b***	***b***	***b***
	***F2-2***	***A2B1***	***a***	***a***	***b***	***b***	***b***	***b***
Basidium F	**F2-3**	**A2B2**	**b**	**b**	**b**	**b**	**b**	**b**
	**F2-4**	**A2B2**	**b**	**b**	**b**	**b**	**b**	**b**
	***F2-5***	***A2B1***	***a***	***a***	***b***	***b***	***b***	***b***
	**F2-6**	**A2B2**	**b**	**b**	**b**	**b**	**b**	**b**

Bold: fertile with parents;

Bold and Italics: fertile with siblings from F1 set 1 and F2 progeny;

Underlined genotypes indicate intra-*MAT* (A or B locus) recombinant progeny;

1 : “a” represents allele from A1B1 parent CBS6039; “b” represents allele from A2B2 parent CBS6273.

**Table 2:** Summary of genotypes of F1 set 2 and F2 progeny using RAPD markers.

	OPA4_MODI	OPA5_MODI	Pi_Random_5	Pi_Random_8	Pi_Random_9	Pi_Random_15	Pi_Random_20	Pi_Random_21	Pi_Random_24_No.1	Pi_Random_24_No.2	JOHE22492	JOHE22621	JOHE22631	JOHE22643	JOHE22655_No.1	JOHE22655_No.2	JOHE22655_No.3	JOHE22656_No.1	JOHE22656_No.2	JOHE22660	% of allels from CBS6039 3	Genotypes 4	Number of genotypes 5
CBS6039^1^	a	a	a	a	a	a	a	a	a	a	a	a	a	a	a	a	a	a	a	a			
CBS6273^2^	b	b	b	b	b	b	b	b	b	b	b	b	b	b	b	b	b	b	b	b			
F1S2_1	a	a	a	b	a	a	b	a	a	b	a	a	a	a	b	a	b	b	b	a	65	A	6
F1S2_2	b	b	b	b	a	a	b	b	b	a	a	a	b	b	b	a	a	b	a	b	40	B	
F1S2_3	b	b	b	a	b	b	a	b	b	b	b	b	a	b	a	b	a	a	b	b	30	C	
FIS2_4	a	b	a	a	b	a	b	a	a	a	a	b	b	a	b	b	b	a	a	a	60	D	
F1S2_5	a	a	a	a	b	b	b	a	a	a	b	b	b	a	a	b	b	a	a	a	60	E	
F1S2_6	a	a	a	a	b	b	b	a	a	a	b	b	b	a	a	b	b	a	a	a	60	E	
F1S2_7	b	b	b	b	a	a	b	b	b	a	a	b	b	b	b	a	a	a	a	b	40	F	
F1S2_8	b	b	b	b	b	a	a	b	b	a	b	b	a	a	b	b	b	a	b	b	30	A	8
F1S2_9	a	a	a	a	a	b	a	a	a	b	a	a	b	b	a	b	b	b	a	a	65	B	
F1S2_10	a	a	a	b	b	a	a	b	b	b	a	b	a	b	a	a	a	b	a	a	60	C	
F1S2_11	b	b	b	b	b	b	a	b	b	a	b	b	a	a	b	a	b	a	b	b	30	D	
F1S2_12	b	b	b	b	b	b	b	b	b	a	b	b	a	a	b	a	b	a	b	b	25	E	
F1S2_13	b	b	b	a	a	b	b	a	a	a	b	a	b	a	b	b	b	a	b	b	40	F	
F1S2_14	b	b	b	b	b	b	b	b	b	a	b	b	a	a	b	a	b	a	b	b	25	E	
FIS2_15	a	a	a	b	b	a	a	b	b	b	a	b	a	b	a	a	a	b	a	a	60	C	
F1S2_16	a	a	a	b	b	a	a	b	b	b	a	b	a	b	b	a	a	b	a	a	55	G	
F1S2_17	b	b	b	a	a	b	b	a	a	a	b	a	a	a	b	b	b	a	b	b	45	H	
F1S2_18	a	a	a	a	b	b	b	a	a	a	b	b	b	a	a	b	b	a	a	a	60	A	5
FIS2_19	a	a	a	b	a	a	a	a	a	b	a	a	a	a	b	a	b	b	b	a	70	B	
F1S2_20	b	b	b	a	b	b	a	b	b	b	b	b	a	b	a	b	a	a	b	b	30	C	
F1S2_21	a	a	a	a	a	a	a	a	a	b	a	a	a	a	a	a	b	b	b	a	80	D	
F1S2_22	a	a	a	a	b	b	b	a	a	a	b	b	b	a	a	b	b	a	a	a	60	A	
F1S2_23	b	b	b	b	a	b	b	b	b	a	a	a	b	b	b	a	a	b	a	b	35	E	
F1S2_24	b	b	b	a	b	b	a	b	b	b	b	b	a	b	a	b	a	a	b	b	30	C	
F1S2_25	a	a	b	b	a	a	a	b	b	a	a	a	b	b	a	a	a	a	b	a	65	A	1
F1S2_26	a	a	b	b	a	a	a	b	b	a	a	a	b	b	a	a	a	a	b	a	65	A	
F1S2_27	a	a	b	b	a	a	a	b	b	a	a	a	b	b	a	a	a	a	b	a	65	A	
F1S2_28	a	a	b	b	a	a	a	b	b	a	a	a	b	b	a	a	a	a	b	a	65	A	
F1S2_29	a	a	b	b	a	a	a	b	b	a	a	a	b	b	a	a	a	a	b	a	65	A	
F1S2_30	a	a	b	b	a	a	a	b	b	a	a	a	b	b	a	a	a	a	b	a	65	A	
F1S2_31	a	a	b	b	a	a	a	b	b	a	a	a	b	b	a	a	a	a	b	a	65	A	
% of alleles from CBS6039^6^	61	58	38	38	52	55	58	35	35	68	58	48	45	45	58	61	52	71	39	61			
F2_1	b	b	b	a	b	b	b	b	b	b	b	b	a	b	b	b	b	b	b	b	10	A	1
F2_2	b	b	b	a	b	b	b	b	b	b	b	b	a	b	b	b	b	b	b	b	10	A	
F2_3	b	b	b	b	b	b	a	b	b	b	b	b	a	b	a	b	a	a	b	b	25	A	3
F2_4	b	b	b	b	b	b	a	b	b	b	b	b	a	b	a	b	a	a	b	b	25	A	
F2_5	b	b	b	a	b	b	a	b	b	b	b	b	a	b	a	b	a	a	b	b	30	B	
F2_6	b	b	b	b	b	b	a	b	b	b	b	b	b	b	a	b	b	a	b	b	15	C	

1 “a” represents CBS6039 allele;

2 “b” represents CBS6273 allele;

3 Percentage of CBS6039 alleles within each progeny at the markers analyzed;

4 Genotypic category based on the markers analyzed in this study for each progeny from the same basidium;

5 Number of unique genotypes among the progeny from the same basidium;

6 Percentage of CBS6039 alleles among the F1S2 progeny at each marker.

### Mating ability of meiotic progeny

**Figure 9:**
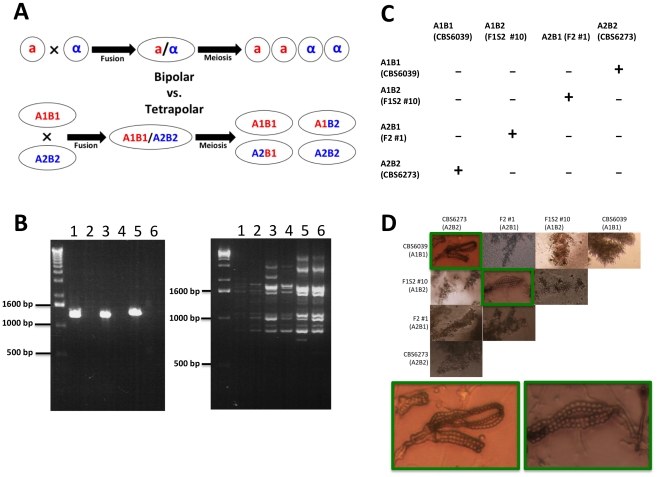
*C. amylolentus* has a tetrapolar mating system. (A) In a bipolar mating system, haploid **a** and α cells fuse to form a diploid **a**/α cell. Sex culminates in meiosis, which gives rise to four meiotic progeny, 2 **a** and 2 α. The **a** progeny can mate with the α parent (50%) while the α progeny can mate with the **a** parent (50%). In a tetrapolar mating system, haploid A1B1 and A2B2 cells fuse to form a dikaryon/diploid A1B1/A2B2. Meiosis then results in the production of four haploid meiotic progeny: A1B1 can mate with the A2B2 parent and progeny (25%), A2B2 can mate with the A1B1 parent and progeny (25%), and A1B2 and A2B1 are recombinants (50%) that are sterile with either parent but interfertile with one another. (B) An example of a RAPD and genotyping marker analysis on four progeny and the two parental strains that represent the different gentoypes in a tetrapolar mating system (1 = F1S2 #3 (A1B1), 2 = F1S2 #13 (A2B2), 3 = F2 #1 (A2B1), 4 = F1S2 #10 (A1B2), 5 = CBS6039 (A1B1), and 6 = CBS6273 (A2B2)). (C) Results of mating assays of all possible combinations among the four mating types. Mating was performed by mixing strains on V8 plate (pH = 5). (“−” indicates lack of sexual reproduction and “+” indicates sexual reproduction occurs). (D) Microscopic images of hyphae and spore chains generated during *C. amylolentus* mating assays described in Figure 9C (the mating-type of each strain is indicated in parenthesis). Dikaryotic hyphae and spore chains were produced in matings between CBS6039 (A1B1) and CBS6273 (A2B2) and between F1 set2 #10 (A1B2) and F2 #1 (A2B1). Monokaryotic hyphae were produced in all of the other mating combinations, including individual strains grown in the absence of a mating partner.

We discovered that some of the progeny that are sterile with either parent are in fact interfertile with other progeny. Specifically, of the 59 F1 progeny (mixture of blastospores and basidiospores), there are 14 A1B1 F1 progeny that are fertile with the A2B2 parent CBS6273, one A2B2 progeny that is fertile with the A1B1 parent CBS6039, and one that is fertile with both parents. Among the spore chain derived (F1S2), progeny #13 (A2B2) was found to be able to mate with progeny #3 and #24 (A1B1). Successful mating was also observed between some *MAT* recombinant progeny. Specifically, successful mating was observed when A1B2 progeny (F1S2 #10 and #16) and A2B1 progeny (F1S1 #18, F2 #1, #2, and #5) were co-cultured together (with the exception of mating between F1S2 #16 and F1S1 #18). None of these *MAT* recombinant progeny mated with either parent, further confirming that *C. amylolentus* possesses a tetrapolar mating system (Figure 9).

Because only 32% (19/59) of the progeny are fertile in both progeny sets, we assessed whether fertility increases with an additional sexual cross or mitotic passage. Even after several passages on YPD, the sterile phenotype remained stable (data not shown). We crossed F1S2 progeny #3 and CBS6273 to generate a backcross progeny set (F2). Interestingly, most of the basidia in the cross were barren and if spore chains were present, the number of spores per chain was significantly reduced when compared to matings between the parental strains. We were successful in dissecting spores from two individual spore chains. The germination rate was 54% (6/11) and all of the progeny were fertile (50% with the CBS6039 parent and the remaining A2B1 progeny are interfertile with the F1S2 progeny #10 and #16 (Table 1)). All of the progeny examined are haploid with the exception of F2 #4, which is diploid by FACS yet remains self-sterile (data not shown).

In summary, taken together our genotyping data indicates that meiotic recombinants are present among the sexually produced progeny and our evidence is that the sexual cycle of *C. amylolentus* conforms to a modified tetrapolar mating system in that 1) sterile progeny are also frequently produced, and 2) the ratio of the four mating types is unbalanced.

### Uniparental mitochondrial DNA inheritance

To assess the mitochondrial inheritance pattern during sexual reproduction of *C. amylolentus*, SNPs were first identified in two mitochondrial genes, *NAD4* and *NAD5*, between the two parental strains, CBS6039 and CBS6273, by PCR amplification and sequencing. Of the 65 progeny screened, no intra- or inter-genic recombination between the two genes was observed, and all of the progeny (with the exception of two from F1S1) typed as the CBS6273 (A2B2) parent (Table S3). The two progeny (F1S1 #13 and #16) that contain the A1B1 mitochondrial genome are likely dissected parental yeast cells, because they also both possessed A1B1 alleles at all of the other markers that were typed. For the other nuclear non-recombinant progeny that type as the A1B1 parent, the fact that they have the A1B1 nuclear genome and the A2B2 mitochondrial genome suggests that they descend from blastospores produced after cell-cell fusion and a result from cytoduction of the CBS6039 nuclear genome and CBS6273 mitochondrial genome. All other progeny that are derived from meiotic basidiospores contained a recombinant nuclear genome paired with the mitochondrial genome exclusively from the A2B2 parent (CBS6273). These results demonstrate that mitochondria are uniparentally inherited from the A2B2 parent during *C. amylolentus* sexual reproduction, similar to *C. neoformans* in which mtDNA is inherited uniparentally from the **a** parent [35], [36], [37], [38].

## Discussion

The current study extends the previous analyses of the *MAT* locus in the pathogenic *Cryptococcus* species to the closest known species, *T. wingfieldii* and *C. amylolentus*. To determine the structure of *MAT* in both species, we cloned and sequenced the HD and P/R loci. Due to their close phylogenetic relatedness [22], characterization of *MAT* has provided key insights into the evolution of *MAT* and revealed important aspects of the transition from an ancestral tetrapolar to a bipolar mating system in *C. neoformans* and *C. gattii* [13], [14], [39], [40].

A previous phylogenetic analysis using a six-gene multi-locus sequencing (MLS) approach identified the most closely related species to the pathogenic *Cryptococcus* species complex [22]. This analysis identified the *sensu stricto* (closely related) and *sensu lato* (more distantly related) species that provide unique vantage points to address questions such as: when and how did the bipolar mating system evolve? And what, if any, affects does the emergence of bipolar mating systems have on the pathogenesis of *C. neoformans* and *C. gattii*? Previous studies on a more distantly related *sensu lato* species, *Cryptococcus heveanensis*, revealed it to be tetrapolar [24]. The key advances presented here provide additional critical insights. First, as *sensu stricto* strains, *C. amylolentus* and *T. wingfieldii* are much more closely related to the pathogenic species *C. neoformans*/*C. gattii* than is *C. heveanensis*; hence the transition to bipolarity in the pathogens was even more recent than could be concluded based on the studies of *C. heveanensis* alone. Second, by providing additional tetrapolar outgroup species, we can conclude that the transition was from tetrapolar to bipolar, not vice versa. Third, the P/R locus is much more expanded in *C. amylolentus* compared to *C. heveanensis*, providing further insights on the evolution of the *MAT* and this key step in the process. Furthermore, the tetrapolar mating system in *C. amylolentus* showed indications of deviation from the classic tetrapolar model in that many *MAT* loci recombinant progeny are sterile and progeny that resemble one parent at the *MAT* loci dominate the progeny population. Moreover, the organization of *MAT* in these sibling species mirrors key aspects (gene acquisitions, chromosomal rearrangements, etc.), which shaped the evolution of the mating-type locus in the pathogenic *Cryptococcus* species complex.

Previous analysis resolved the phylogeny surrounding the pathogenic *Cryptococcus* species cluster and revealed that *T. wingfieldii* and *C. amylolentus* are sibling species, the closest relatives of the pathogenic species, and members of the *Filobasidiella* clade [22]. The *MAT* loci of *T. wingfieldii* and *C. amylolentus* share overall synteny, with two major inversion events present between the P/R loci of the two species (Figure 3). For this analysis, the type strain, the only isolate of *T. wingfieldii* available, was employed. Two strains of *C. amylolentus* are available and we characterized *MAT* for the type strain CBS6039 and representative sequences for CBS6273. The two *MAT* loci of *T. wingfieldii* and *C. amylolentus* are physically unlinked and present on different chromosomes (Figure 1B and Figure 2B). The *MAT* assembly for *C. amylolentus* is similar to *T. wingfieldii* in that both homeodomain transcription factors are present and opposite in their orientations, similar to the paired, divergently oriented *bE* and *bW* genes in *U. maydis*. Several other key genes (*SPO14*, *RPL22*, and *CAP1*) are present and these lie within *MAT* in *C. neoformans* but appear to lie outside of *MAT* in *C. amylolentus*. The configuration of the HD genes in the sibling species provides evidence that the ancestral form of the HD locus contained both *SXI1* and *SXI2*, similar to tetrapolar mating systems in other basidiomycetes, and that loss of one or the other of the HD genes punctuated the formation of a bipolar mating system.

Of the >20 genes identified in the HD (*B*) and P/R (*A*) loci of the sibling species, we determined which genes define *MAT*. Because only one strain of *T. wingfieldii* is available, we were unable to establish which of the genes in the *B* and the *A* loci are *MAT*-specific. By comparing sequences from the two strains of *C. amylolentus*, we determined that the *MAT*-specific region in the HD locus is likely restricted to the ∼3 kb *SXI1* and *SXI2* dimorphic region. The divergence is present in the 5′ regions of *SXI1* and *SXI2*, similar to recent findings on the *B MAT* locus alleles of *C. heveanensis* [24]. This is also consistent with findings in other fungi where the N-terminal regions of the homeodomain proteins are typically variable and heterodimerization only occurs when compatible (or different allelic versions) of the proteins are brought together promoting activation of genes required for sexual development [30], [41].

We also sought to define the extent of the sex-specific region in the *MAT A* locus. Our extensive Southern and PCR analysis document that the P/R locus has been expanded to encompass >60 kb in *C. amylolentus*, including the *STE3* and *MF* pheromone genes that lie >30 kb apart in contrast to their close linkage in the P/R *MAT A* locus of *C. heveanensis* (Figures S2, S3, S4). In addition, several genes encompassed within this expanded *C. amylolentus* P/R locus are linked to but outside the defined P/R locus of *C. heveanensis* [24]. Thus, one of the two *MAT* loci has expanded in *C. amylolentus* but the two remain unfused.

We also report the discovery of sexual reproduction in *C. amylolentus*. Fortunately, the only two strains of *C. amylolentus* available in the world are of opposite mating-type and fertile, enabling us to define the sexual cycle for *C. amylolentus*. Mating structures in *C. amylolentus* resemble those observed in *C. neoformans*, and differed from *C. heveanensis*, consistent with its closer phylogenetic relationship with *C. neoformans* than with *C. heveanensis*.

Mating in *C. amylolentus* produces many sterile progeny, suggesting that sexual reproduction may pose a risk in which not all of the progeny produced are fertile. Although the underlying mechanism(s) causing sterility in the *C. amylolentus* progeny is not clear, there are several possible explanations. First, it is possible that aneuploids (1N+1) are generated during meiosis that could be sterile. FACS analysis of the examined progeny suggested that all of the progeny are haploid with the exception of a single diploid (F2 progeny #4), but FACS is not sensitive enough to detect 1N+1 aneuploids. Employing comparative genomic hybridization (CGH) of the *C. amylolentus* parental strains with the sterile progeny will be necessary to address the issue of possible aneuploidy generated during mating. Second, meiosis is mutagenic and sexual reproduction may also increase transposition in the genome. The resulted mutations and/or the insertion of transposons in *MAT* or elsewhere might result in sterility. Third, the increased sterility among progeny could be due to sex induced silencing of repetitive elements within *MAT* and linked fertility genes [42] or damage to *MAT* caused by gene conversion events. Sex induced silencing requires the RNAi machinery. However it is not known yet if *C. amylolentus* possesses these genes. The *C. amylolentus* genome sequence will allow this question to be answered. Additionally, we cannot exclude the possibility that the sterility observed among the progeny is due to divergence/incompatibility between the mating machineries of the two *C. amylolentus* strains, or to nuclear-mitochondrial incompatibility that has been observed in other yeasts [43].

From the genotyping analysis, it is evident that extensive recombination occurred among the progeny produced by sexual reproduction. Additionally, we observed a 1∶1 segregation pattern of the two parental alleles in the progeny population. This segregation data and the high level of genetic exchange in the progeny (especially the F1S2 spore chain derived progeny set) provide strong evidence that meiosis occurs within the basidium during sexual reproduction. Additionally, RAPD analysis revealed that in some spore chains from the F1S2 and the F2 progeny sets, more than four genotypes are present in a single chain. There are several possible explanations. In *C. neoformans*, meiosis typically gives rise to four meiotic products and it was recently shown that a single meiotic event occurs in each basidium [44]. In *C. amylolentus*, more than one meiotic event could occur in the basidium. However, this would have to involve post meiotic nuclear fusion and a second round of meiosis. In this case, up to eight genotypes could be produced from one basidium. Also, high gene conversion events favoring some alleles over others could result in a non-Mendelian inheritance pattern and skew the resulting genotypes in each individual spore chain.

Another possible explanation for the observed >4 genotypes/basidium that we favor is the presence of aneuploids in the progeny population. The RAPD markers employed in this analysis differentiate the two parental strains by the presence or absence of a PCR product. If progeny are aneuploid for one or more chromosome, they could appear unique and differ from the two parental strains. In this aneuploidy model we expect the basidiospores from one basidium to share four common genotypes with the exception of a few rarer genotypes (potential aneuploids). This model is consistent with our RAPD data (Table 2 and Table S2) in which several spore chains contain four distinct major genotypes and several anomalous minority genotypes that are closely related to one of the four consensus majority genotypes in a given spore chain (Figure S9). One limitation is that we are currently unable to score the heterozygous state of the aneuploids, which can be detected by co-dominant markers such as PCR-RFLP and CGH, and this provides fertile ground for future studies.

Micromanipulation of the individual spore chains representing F1S2 and the F2 progeny generated progeny that are recombinant at the *MAT* loci (A1B2 and A2B1), and these *MAT* recombinant progeny are inter-fertile, but cannot mate with either of the two parental strains, proving that *C. amylolentus* has a tetrapolar mating system. However, among those isolates that were fertile the A1B1 genotype was overrepresented, whereas the other three genotypes were underrepresented (Table 1 and Table S1).

*MAT* in the pathogenic *Cryptococcus* species evolved from an ancestral tetrapolar system with physically unlinked *B* and *A* loci and these loci fused into a large bipolar *MAT* locus. In *C. amylolentus*, the structure of *MAT* indicates a tetrapolar mating system with allelic diversity in both the *B* and *A* loci. Although evidence from *T. wingfieldii*, *C. amylolentus*, *C. heveanensis* [24], and *C. disseminatus* [9] suggests that *MAT* evolved from a tetrapolar to a bipolar system, an alternative hypothesis could be just the opposite: namely that the ancestral form of *MAT* was bipolar and instead evolved into a tetrapolar mating system in these species. In such a scenario, a bipolar locus could have suffered a chromosomal break resulting in the formation of physically unlinked HD and P/R loci in a derived rather than ancestral tetrapolar fungal species. In this model, the tetrapolar state would then be ancestral in some species and derived in others. We do not favor this alternative model and the one we propose (Figure 5) instead illustrates the evolution of the bipolar *MAT* in the pathogenic *Cryptococcus* species from an ancestral tetrapolar system. The evidence adduced now for three sibling species supports the more parsimonious model that *C. amylolentus*, *T. wingfieldii*, and *C. heveanensis* all reflect a common, shared ancestral tetrapolar state rather than multiple independent derived states.

*MAT* evolution in fungi has defined a continuum of transitions in modes of sexual reproduction from outcrossing tetrapolar multiallelic systems to bipolar biallelic systems that promote inbreeding to unipolar uniallelic same-sex mating that promotes extreme inbreeding and clonality [15], [45]. Aside from bipolar and tetrapolar mating systems, some deviations from these classic mating systems have been reported recently. For example, a pseudo-bipolar mating system has been recently found in the red yeast *Sporidiobolus salmonicolor* [46], [47]. The authors found that in this species, mating is normally bipolar and governed by a large continuous *MAT* locus with the A and B regions located at either end. However, meiotic recombination may occur between the *MAT* locus alleles, generating novel mating types, and thus increasing *MAT* allele number and evolutionary rates for some *MAT* genes.

Results from our studies illustrate features of both the transition from tetrapolarity to bipolarity in the closely aligned saprobic and pathogenic *Cryptococcus* species, and also the emergence of sexual reproduction in which one mating-type has an advantage resulting in a higher proportion of fertile progeny of one mating-type (A1B1) that might have ultimately led to the emergence of unisexual same-sex mating in *C. neoformans*. In conclusion, *C. amylolentus* and *C. heveanensis* have physically unlinked HD and P/R loci and this arrangement further supports tetrapolarity as the ancestral configuration, and that the transition to bipolarity occurred recently and concomitantly with the emergence of the pathogenic *C. neoformans/C. gattii* species cluster.

These studies on the molecular events leading to the fusion of two unlinked sex determining regions of the genome in the ancestral tetrapolar state to the derived bipolar mating systems mirror aspects in the hypothesized origin of sex chromosomes of more complex multicellular eukaryotes, including plants, insects, fish, and mammals. Namely, Ohno hypothesized that sex determinants arise on an autosome, and then gradually capture this chromosome, which evolves to become a sex chromosome [39]. These steps include the original emergence of the sex determinant, the recruitment of other genes that function in sex to the incipient sex chromosome, and rearrangements and the acquisition of repetitive elements that lead to two sexually dimorphic chromosomes. The transition from two unlinked sex determinants in tetrapolar fungi to two linked sex determinants in bipolar fungi, and the fact that this transition has occurred repeatedly and independently, provides further support for the hypothesis that sex determinants arise at distant genomic locations and then become linked through gene movement or chromosomal translocations in both mating type loci and sex chromosomes.

## Materials and Methods

### Strains and media

The two strains of *C. amylolentus*, CBS6039 and CBS6273, and the one *T. wingfieldii* isolate, CBS7118, were obtained from the Centraalbureau voor Schimmelcultures (CBS) Fungal Biodiversity Centre in the Netherlands. Both CBS6039 and CBS6273 were originally isolated from insect frass in South Africa, while CBS7118 was originally isolated from rubber sheet in Indonesia. All species were grown and maintained on yeast extract-peptone-dextrose (YPD) medium at 24°C. Mating assays were performed on V8 medium pH = 5 in the dark and also at 24°C. Random spore dissection was performed on YPD medium as previously described [44]. Spore chain dissection was performed by first transferring a well separated spore chain onto a drop of zymolyase on YPD, and after incubation at 24°C for 15 minutes, individual spore from the spore chain was dissected as previously described [44].

### DNA extraction

To isolate genomic DNA from *T. wingfieldii* and *C. amylolentus*, cells were cultured in 50 ml of liquid YPD shaking overnight at 24°C. The pellets were then lyophilized overnight and the CTAB method of fungal DNA isolation was performed as described before [24]. Plasmid DNA from positive TOPO clones was extracted using the QIAprep Spin Miniprep Kit (Qiagen, Valencia, CA), fosmid DNA was isolated using a modified miniprep protocol, and DNA from the shot-gun sequencing libraries was extracted using the DirectPrep96 Miniprep Kit (Qiagen, Valencia, CA). Additionally, progeny DNA was isolated using a modified miniprep protocol and colony lifts were performed to isolate DNA from individual colonies in each fosmid library according to the protocol described in [28].

### Degenerate PCR

We designed degenerate PCR primers using the online computer program, COnsensus-DEgenerate Hybrid Oligonucleotide Primer (CODEHOP, http://blocks.fhcrc.org/codehop.html) to identify *MAT* specific genes in *T. wingfieldii*. The primers consist of a relatively short 3′ degenerate core and a longer 5′ non-degenerate consensus clamp designed by multiple sequence alignments [48]. We aligned sequences for two flanking genes (*FAO1* and *NOG2*) and two recently acquired *MAT* genes (*RPO41* and *LPD1*) from *C. neoformans* var. *neoformans* and var. *grubii*, *C. gattii*, *U. maydis*, and *C. cinerea* to design the degenerate PCR primers (see Table S4 for primer information). PCR was performed on genomic DNA isolated by the CTAB extraction method as template and products were separated by gel electrophoresis. Products with the strongest ethidium bromide-staining signal were then gel extracted using the QIAquick Gel Extraction Kit (Qiagen, Valencia, CA) followed by transformation into *E. coli* using the TOPO-TA cloning Kit (Invitrogen, Carlsbad, CA). Plasmid DNA was purified from transformants and then sequenced. For *C. amylolentus*, degenerate primers were not used. Instead, primers from *T. wingfieldii* were directly used to amplify *MYO2*, *LPD1*, *SXI1*, and *SXI2* in both *C. amylolentus* strains (see Table S4 for primer information).

### Fosmid library preparation and fosmid library screening

We employed the CopyControl Fosmid Library Production Kit (Epicentre, Madison, WI) to generate fosmid libraries for *T. wingfieldii* and *C. amylolentus* strain CBS6039. At least 2.5 µg of CTAB isolated genomic DNA was randomly sheared using a 200 µl small bore pipette tip and sheared DNA was end-repair converted to blunt 5′ phosphorylated ends using End-Repair Enzyme Mix, dNTPs, and ATP. We then separated the end-repaired DNA overnight using a Contour-clamped Homogenous Electric Field (CHEF) on a CHEF DR-II apparatus (Bio-Rad, Hercules, CA). The following conditions were used: 1- to 6-second switch time, 6 V/cm, 14°C for 14–15 hrs in 0.5X TBE. The size-fractionated DNA, 25 to 40 kb fragments, was recovered by gel extraction and the DNA was precipitated with sodium acetate and ethanol. The precipitated insert DNA was then ligated into the CopyControl pCC1FOS cloning-ready vector and incubated overnight at 24°C. The ligated DNA was packaged in phage particles and plated on *E. coli* phage-resistant cells (EPI100-T1R plating strain) overnight at 37°C (detailed protocol can found at http://www.epibio.com/item.asp?ID=385). Approximately 16,000 fosmid clones were picked into 96-well plates and transferred to 384-well plates for long-term storage at −80°C. The 384-well plates were replicated onto high-density filters for hybridizations using the *MAT* genes.

### Sequencing and assembly

Positive fosmid clones were sequenced using the shot-gun sequencing method described by Metin et al. [24]. Six fosmids were pooled and sequenced to generate the assembly for *T. wingfieldii* and four fosmids were individually sequenced to generate the assembly for *C. amylolentus* strain CBS6039. Sequencing reactions were performed using Big Dye chemistry v3.1 (Applied Biosystems, Foster City, California, United States) and analyzed on an Applied Biosystems 3730xl capillary sequencer in the Biological Sciences Sequencing Facility at Duke University. For each library, approximately ∼1200 sequence reads were imported into UNIX using Phred and Phrap to assemble the sequences into larger contigs of overlapping sequence [49], [50], [51]. To close gaps in the assemblies, we designed primers from contig ends using Primer 3 (http://frodo.wi.mit.edu/primer3/). The GenBank accession numbers for *T. wingfieldii* are HM368525 (HD locus) and HM368524 (P/R locus). The GenBank accession numbers for the HD locus and the three P/R contigs in *C. amylolentus* CBS6039 are: HM640220 (HD locus), HM640221 (*RPL39-MYO2*), HM640222 (*LPD1-STE12*), and HM640223 (*GEF1-MFA*). The GenBank accession numbers for genes from *C. amylolentus* CBS6273 are: HM640224 (*SXI1*), HM640225 (*SXI2*), HM640226 (*GEF1*), HM640227 (*LPD1*), and HM640228 (*ETF1*).

### Fluorescence-activated cell sorting (FACS) analysis

To determine the ploidy of the two *C. amylolentus* and one *T. wingfieldii* strains, we cultured the isolates on YPD medium for 2 days at 24°C. Each isolate was processed for flow cytometry as previously described [5], [52] and analyzed using the FL1 channel on a Becton-Dickinson FACScan. The ∼20 Mb genome of *C. neoformans/gattii* was used as a reference for ploidy determination (including haploid and diploid controls).

### Pulsed-field gel electrophoresis (PFGE) and chromoblot analysis

To isolate chromosomal DNA of *C. amylolentus* and *T. wingfieldii*, spheroplasts were generated following the spheroplasting protocol for *C. neoformans* and *C. gattii* [53]. The plugs containing spheroplasts were lysed at 55°C for at least 24 hrs in lysing solution (0.5 M EDTA/10 mM Tris-Cl (pH = 10) and 1% Sarcosyl) and then loaded onto a PFGE apparatus and separated for approximately 5 days on a CHEF DR-II apparatus (Bio-Rad, Hercules, CA). The following conditions were used: Block 1: 75- to 150-second switch time, 4 V/cm, 13°C for 30 hrs and Block 2: 200 to 400-second switch time, 4 V/cm, 13°C for 60 hrs in 0.5X TBE. The gel was then stained in ethidium bromide for 15 minutes, destained for an hour, and visualized using a UV lamp. The chromosomal DNA was blotted overnight onto Hybond (Amersham, Piscataway, NJ) membranes in 20X SSC using standard protocols. The membrane was then hybridized to *MAT* gene probes generated by PCR. We also performed Southern blot analysis on genomic DNA from *C. amylolentus* that was digested with EcoRV, PstI, BamHI, or NotI. The digested DNA was separated on an agarose gel and probed with the *RPL22* gene probe amplified from *C. amylolentus*, with primers designed for *T. wingfieldii* (see Table S4 for primer information).

### Bioinformatic analyses

We compared sequences from the HD locus of *T. wingfieldii* to those of *C. amylolentus* by employing a matrix comparison (or dot plot) analysis. To generate each dot plot, we employed the Molecular Toolkit's online nucleic acid dot plots program (http://www.vivo.colostate.edu/molkit/dnadot/). The parameters for the dot plot analyses were as follows: the window size was 51 and the mismatch limit was 6. We also employed the bioinformatic software, Artemis Comparison Tool Release 8 (http://www.sanger.ac.uk/resources/software/) to generate comparison plots across *MAT* of *T. wingfieldii* to *C. amylolentus* and both sibling species compared to *C. neoformans* serotype D strain JEC21 [54]. The input file was created using WebACT (http://www.webact.org/WebACT/home) with the Blastn algorithm [55].

### Phylogenetic analysis

Phylogenetic analysis was performed on coding sequences using MEGA 5 [56]. To determine the phylogenetic relationship, the Neighbor-Joining method based on the Kimura 2-parameter model was employed [57]. For statistical support, 500 replicates were performed and bootstrap values were calculated.

### Southern blot analysis

We performed Southern blot analysis using standard protocols on genomic DNA from *C. amylolentus* digested with BamHI, BglI, ClaI, EcoRI, or NcoI. The digested DNA was separated on an agarose gel and probed with the *STE3* gene, stripped (0.1% SDS and 0.1X SSC in boiling water, 3 times for 15 minutes each), and probed with the contig ends from the P/R assembly in *C. amylolentus* amplified by PCR (see Table S4 for primer information).

### Description of the sexual cycle as *Filobasidiella amylolenta*

Standard description: *Filobasidiella amylolenta* Findley & Heitman sp. nov.

Etymology: The epithet is chosen to be identical with that of *C. amylolentus* (Van der Walt, D.B. Scott & Klift) Golubev 1981 [58]. Heterothallic fungus. Hyphae dikaryotic, clamped connections fused. Aseptate basidia, 3–5 µm diameter, terminating in four chains of basidiospores. Basidiospores are aerial, round, and 2–2.5 µm in diameter.

Holotype: Mounted teleomorph is paired cultures of *C. amylolentus* type strain, CBS6039^T^ (A1B1) crossed to CBS6273 (A2B2) on V8 medium (pH = 5). These strains were originally isolated from insect frass in South Africa [58]. A slide preparation of mating structures, basidia and basidiospores, is deposited in the USDA's Systematic Mycology and Microbiology Laboratory in Beltsville, Maryland (deposit number: BPI 881008). Strains CBS6039 (mating-type A1B1) and CBS6273 (mating-type A2B2) should be designated as the ex-type strain and the isotype strain, respectively, for the teleomorph *Filobasidiella amylolenta*.

Latin description: *Filobasidiella amylolenta* Findley & Heitman sp. nov.

Fungus heterothallicus. Hyphae dikaryoticae, fibulis fusis. Basidia aseptata, 3–5 µm lata, quatuor catenas basidiosporarum producentia. Basidiosporae aeriae, globosae, 2–2.5 µm diametro.

### Microscopy

Spores and yeast cells were cultured on slides coated with V8 pH = 5 medium for one week or longer to allow production of mating structures. The slide was first washed with phosphate buffered saline (PBS) followed by staining the cell wall using a solution of Calcofluor white (fluorescent brightener 28 F-3397; Sigma) for 15 minutes. Slides were rinsed with PBS and fixed for 15 minutes in fixing solution (3.7% formaldehyde and 1% Triton-X100 in PBS). After permeabilization of the fungal cells, nuclear content was examined by staining with Sytox green (Molecular Probes) for 30 minutes. Slides were washed with PBS and a cover slip was applied to the slide for observation. In addition to staining spores and yeasts, mating filaments were also stained. Agar pieces were removed from mating plates and washed several times with PBS. Calcofluor white was added directly to the agar piece for 30 minutes, followed by washing with PBS, and fixing for 45 minutes. After permeabilizing samples, filaments were washed with PBS and stained with 1 mg/ml Hoechst 33258 (Invitrogen, Carlsbad, CA) overnight at 4°C. The next day, samples were washed with PBS, a thin slice of the agar (containing the mating filaments) was removed using a razor blade and a mounting solution containing anti-fade (Invitrogen, Carlsbad, CA) was added to the agar slice on a slide. The slides were sealed with nail polish and stored at 4°C in the dark after microscopic evaluation. All staining was performed at 24°C, unless otherwise noted. SEM was performed on *C. amylolentus* matings incubated on V8 pH = 5 medium for 2 weeks. The specimen was prepared and analyzed as described in [28]. Microscopy was performed with an Axioskop 2 plus upright microscope (Zeiss). Images were captured using an AxioCam MRm camera. Scanning electron microscopy was performed and viewed on a JEOL JSM 5900LV (JEOL U.S.A., Peabody, MA) SEM at 15 kV.

### Spore dissection and genotyping

Microdissection of spores (random or individual spore chains using zymolyase (Zymo Research Corp., Orange, CA, USA)) was performed on YPD medium incubated at 24°C for two days to allow spores to germinate.

Genotyping of the *MAT* loci was achieved using a set of PCR markers (*RPL39*, *GEF1*, and *STE3*) and PCR-RFLP markers (*SXI1* (enzyme EcoRV), *SXI2* (enzyme RsaI), and *ETF1* (enzyme DdeI)). To genotype other genomic regions, we used a set of 20 RAPD markers (Table S4). Linkage analyses indicated 18 of these 20 markers are not derived from the *C. amylolentus MAT* loci, with exception of markers Pi_Random_24_No.2 and JOHE22656_No.1, which were positioned in the same linkage group with HD markers. Non-*MAT*-association of nine of these 18 markers were further confirmed by cloning and sequencing of the polymorphic bands, as none of them was *MAT* specific sequence (data not shown). We designate the CBS6039 parent as A1B1 and the CBS6273 parent as A2B2, according to the designation used for a tetrapolar mating system and our findings, assigning *A* as the P/R locus and *B* as the HD locus (as in *T. mesenterica*, *C. heveanensis*, and *U. maydis* [24], [26]). Recombination was scored according to marker exchange for the P/R and/or HD locus. Recombination frequency among RAPD markers was inferred using program MapMaker.

These genotyping data was further analyzed using program MapMaker to generate genetic linkage groups. Additionally, the UPGMA clustering method implemented in the software MEGA 5 was used to analyze the genetic relationships among F1S2 progeny isolated from the same basidium.

## Supporting Information

Figure S1 Fosmid map of the HD and P/R assembly in *T. wingfieldii*. Two overlapping fosmids (2B23 and 2K10) constituting the HD locus, three overlapping fosmids (3F11, 3A15, and 5J15) constituting the P/R locus, and a separate fosmid (4E07) containing the gene *FAO1* and unlinked to either the HD or P/R locus, were sequenced. The *MAT* loci are embedded within regions spanning a total ∼110 kb.
(TIF)

Figure S2 Fosmid map of the HD and P/R assembly in *C. amylolentus*. One fosmid (4E01) constitutes the HD locus while three fosmids (3N14, 4E22, and 3H19) span the P/R locus. The regions containing the *MAT* loci represent ∼80 kb in total. Southern blots are shown in the bottom half of the figure. This data supports the current assembly of the *C. amylolentus* P/R locus shown above. In blots (a), (b), and (e), genomic DNAs from CBS6039 and CBS6273 were digested with BamHI, while for blots (c), (d), and (f) DNA was digested with ClaI (left) or EcoRI (right). The probes used for Southern blot analysis were located at the ends of the three contigs (indicated with the red block arrows). Sizes of the restriction fragments are also indicated. For probes (a), (b), (c), and (e), no hybridization signals were detected for CBS6273, indicating high levels of nucleotide polymorphism between CBS6039 and CBS6273 at these regions. Probe labels 1L, 1R, 2R, 3L, and 3R correspond to those in Figure S3.
(TIF)

Figure S3 Estimations of the sizes of the gaps within the PR locus assembly based on Southern blotting. The numbers in the parentheses indicate the locations of the restriction enzyme recognition sites within their respective contigs. Block arrows show the locations of the probes used for Southern blotting (see images in Figure S2). The numbers below the dotted lines are the size estimates of the fragments produced from digestions by restriction enzymes. The gap sizes were calculated by subtracting the size of the digestion fragment with the sequences obtained within the fragment interval.
(TIF)

Figure S4 Results of PCR assay indicating similarity and divergence between CBS6039 and CBS6273 within the P/R locus assembly. Primers were designed based on the CBS6039 P/R locus assembly, and were used for PCR reactions using either CBS6039 or CBS6273 genomic DNA as template. Black squares indicate primer pairs that produced PCR products in both strains; gray squares indicate primer pairs that yielded PCR products for CBS6039 but not for CBS6273; white squares indicate primer pairs that yielded no PCR product for either strain. Genes from the CBS6039 P/R locus assembly are labeled at the top. Gaps No.1 and No.2, as well as the contig end labels (1L, 1R, 2L, 2R, 3L, and 3R) correspond to those in Figures S2 and S3.
(TIF)

Figure S5 Extensive chromosomal rearrangements are present throughout *MAT* in the sibling species and *C. neoformans* strain JEC21. *MAT* sequences from *T. wingfieldii* and *C. amylolentus* were compared to *C. neoformans* and a synteny analysis was performed. Red denotes conserved gene order while blue indicates inversion events. Green bars under the assembly denote gaps in assembly.
(TIF)

Figure S6 Phylogenetic analysis of additional *C. amylolentus* genes. The phylogenetic relationship of *C. amylolentus* and *T. wingfieldii* to the pathogenic *Cryptococcus* species and neighboring taxa is highlighted and four representative genes *GEF1*, *CID1*, *STE12*, and *ETF1* are shown. *GEF1* and *CID1* exhibit a species-specific phylogeny in *C. neoformans* and *C. gattii*, while *STE12* and *ETF1* exhibit mating-type specific phylogeny. The trees were constructed using the Neighbor-Joining method implemented in the software MEGA4. Bootstrap values on tree branches were calculated from 500 replicates. (α) indicates strains with the *MAT*α locus, and (a) indicates strains with the *MAT*a locus.
(TIF)

Figure S7 Phylogenetic analysis of the homeodomain transcription factor genes, *SXI1* and *SXI2* in *C. amylolentus*. The *SXI1* and *SXI2* genes were analyzed in *C. amylolentus* and neighboring taxa and the gene trees were constructed using the Neighbor-Joining method implemented with the software program MEGA4. Bootstrap values on tree branches were calculated from 500 replicates. (α) indicates strains with the *MAT*α locus, and (a) indicates strains with the *MAT*a locus.
(TIF)

Figure S8 Fluorescence microscopy of *C. amylolentus* mating structures. (A and B) Staining of basidiospores, scale bars = 2 µm. (A) Differential Interference Contrast (DIC) image, and (B) fluorescence image of basidiospores nuclei stained with Hoechst 33258. (C and D) Staining of a dikaryotic mating filament. (C) DIC image, and (D) fluorescence image of mating filaments, in which nuclei were stained with Hoechst 33258. (E–F) Staining of mating filament and clamp cell. (E) Nuclear content of filament stained with Sytox green and (F) Calcofluor white for cell wall visualization. (G) Nuclear content of basidiospores stained with Sytox green and (H) cell wall with Calcofluor white (scale bar = 5 µm).
(TIF)

Figure S9 Evidence for one meiotic event in each basidium. Three basidia were analyzed: basidium A includes F1S2 progeny 1 to 7; basidium B includes F1S2 progeny 8 to 17; basidium C includes F1S2 progeny 18 to 24. The genetic distances were calculated using the UPGMA method implemented with the MEGA 5 program. For all three basidia, each harbors four major clusters of consensus progeny genotypes. Some clusters have several progeny that are closely related yet slightly differ from a majority genotype. This is consistent with a scenario in which one meiosis event occurred during sexual reproduction, and some atypical progeny are aneuploid at a few genetic loci from the consensus meiotic genotypes.
(TIF)

Figure S10 The *SXI1* and *SXI2* dimorphic region is similar in CBS6039 and F1 set 2 progeny #4. Percent identity plots comparing the ∼2 kb region containing the HD genes in the *C. amylolentus* parental strain CBS6039 compared to set 2 F1 progeny #4.
(TIF)

Figure S11 F1 Set 2 progeny #16 is recombinant and types as CBS6039 at *SXI1* and as CBS6273 at *SXI2*. RFLP analysis of the recombinant F1S2 progeny #16. The progeny and both parental strains were digested with EcoRV and RsaI at *SXI1* and *SXI2*, respectively. This analysis reveals a short gene conversion track or very local double crossover event resulted in a novel B *MAT* locus marker allele. These enzymes only cleave the PCR product in the parental strain CBS6039. L = 1 kb ladder.
(TIF)

Figure S12 The *SXI1-SXI2* dimorphic region differs between CBS6273 and progeny set 2 F1 progeny #16. Percent identity plots comparing the ∼2 kb region containing the HD genes in the *C. amylolentus* parental strain CBS6273 compared to set 2 F1 progeny #16. An example of the region of crossover or gene conversion in the HD locus of the recombinant progeny #16 is highlighted with an orange box.
(TIF)

Figure S13 Mating assays between *MAT* recombinant progeny and the bi-mater set 1 F1 #4 crossed to the parental strains. Light microscopy of mating structures produced in the interfertile progeny (top row, Left panels - F1 set 2 #10×F2 #1 and right panels - F1 Set 2 #16×F2 #1 and F1 Set 2 #16×F2 #5) and backcrosses of progeny #4 to CBS6039 and CBS6273 (bottom row). Scale bar = 10 µm. Mating assays were performed as described in the Materials and Methods. Briefly, the two strains were mixed, and the mixture was spotted onto a V8 (pH = 5) medium plate and incubated in the dark at room temperature for four weeks.
(TIF)

Table S1 Random spore dissection of set 1 F1 progeny and molecular analysis of the nuclear markers.
(DOCX)

Table S2 RAPD analysis of F1 set 1 progeny.
(DOCX)

Table S3 List of primers, RAPD markers, and mitochondrial markers used in the study.
(DOCX)

Table S4 Filamentous phenotype and mitochondrial DNA (mtDNA) inheritance identified in all 65 progeny.
(DOCX)

Text S1 Additional discussions on strains, filamentous phenotype, *RPL22* duplication, and analyses of HD dimorphic region in key progeny.
(DOCX)
