# Supplementary figures and images for: Discovery of a Modified Tetrapolar Sexual Cycle in Cryptococcus amylolentus and the Evolution of MAT in the Cryptococcus Species Complex
Source: PLoS Genet. 2012 Feb 16;8(2):e1002528. doi: 10.1371/journal.pgen.1002528 (PMC3280970; doi:10.1371/journal.pgen.1002528)

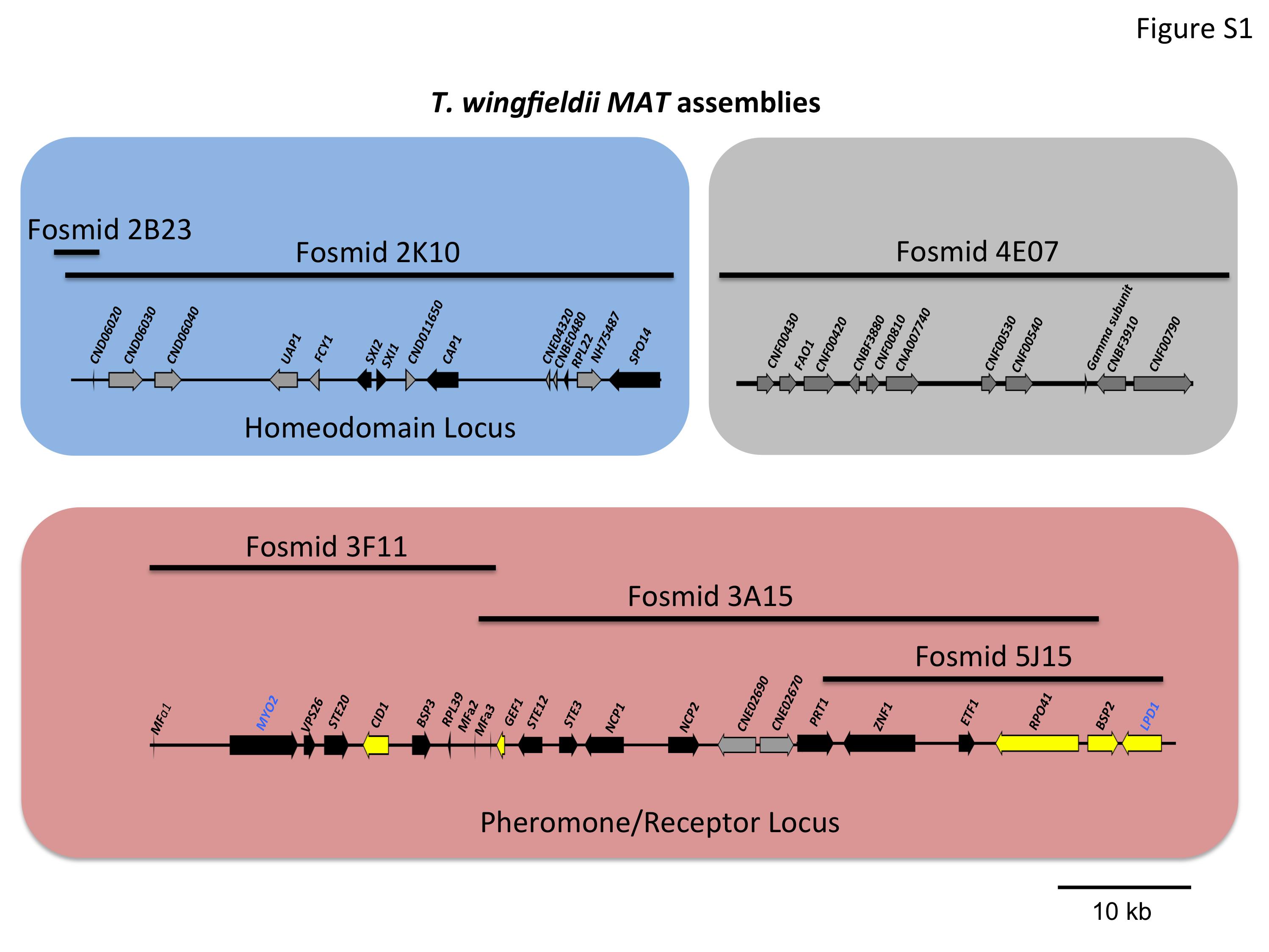

Supplement: Figure S1 — Fosmid map of the HD and P/R assembly in T. wingfieldii. Two overlapping fosmids (2B23 and 2K10) constituting the HD locus, three overlapping fosmids (3F11, 3A15, and 5J15) constituting the P/R locus, and a separate fosmid (4E07) containing the gene FAO1 and unlinked to either the HD or P/R locus, were sequenced. The MAT loci are embedded within regions spanning a total ∼110 kb. (TIF) [file pgen.1002528.s001.tif]

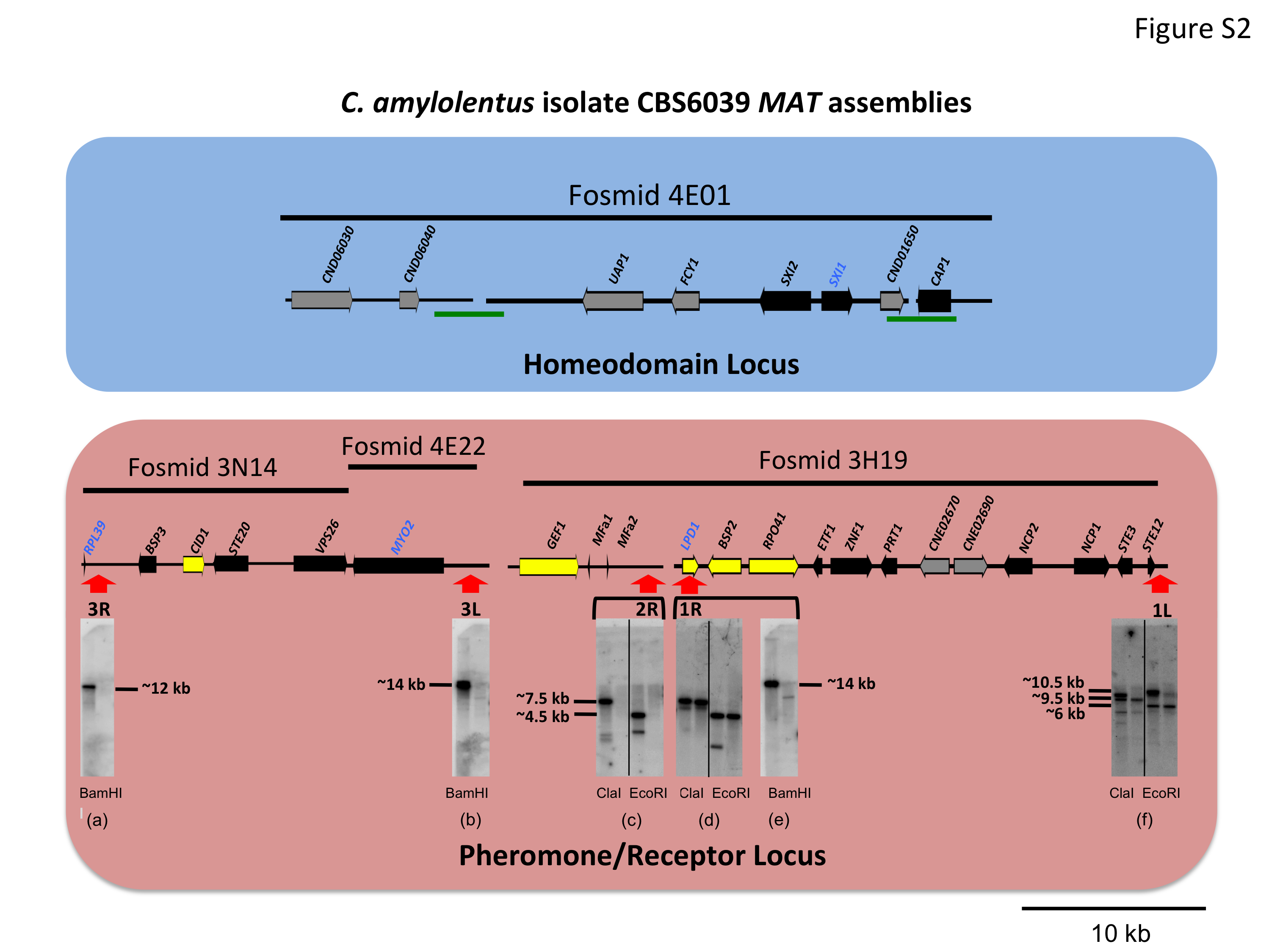

Supplement: Figure S2 — Fosmid map of the HD and P/R assembly in C. amylolentus. One fosmid (4E01) constitutes the HD locus while three fosmids (3N14, 4E22, and 3H19) span the P/R locus. The regions containing the MAT loci represent ∼80 kb in total. Southern blots are shown in the bottom half of the figure. This data supports the current assembly of the C. amylolentus P/R locus shown above. In blots (a), (b), and (e), genomic DNAs from CBS6039 and CBS6273 were digested with BamHI, while for blots (c), (d), and (f) DNA was digested with ClaI (left) or EcoRI (right). The probes used for Southern blot analysis were located at the ends of the three contigs (indicated with the red block arrows). Sizes of the restriction fragments are also indicated. For probes (a), (b), (c), and (e), no hybridization signals were detected for CBS6273, indicating high levels of nucleotide polymorphism between CBS6039 and CBS6273 at these regions. Probe labels 1L, 1R, 2R, 3L, and 3R correspond to those in Figure S3. (TIF) [file pgen.1002528.s002.tif]

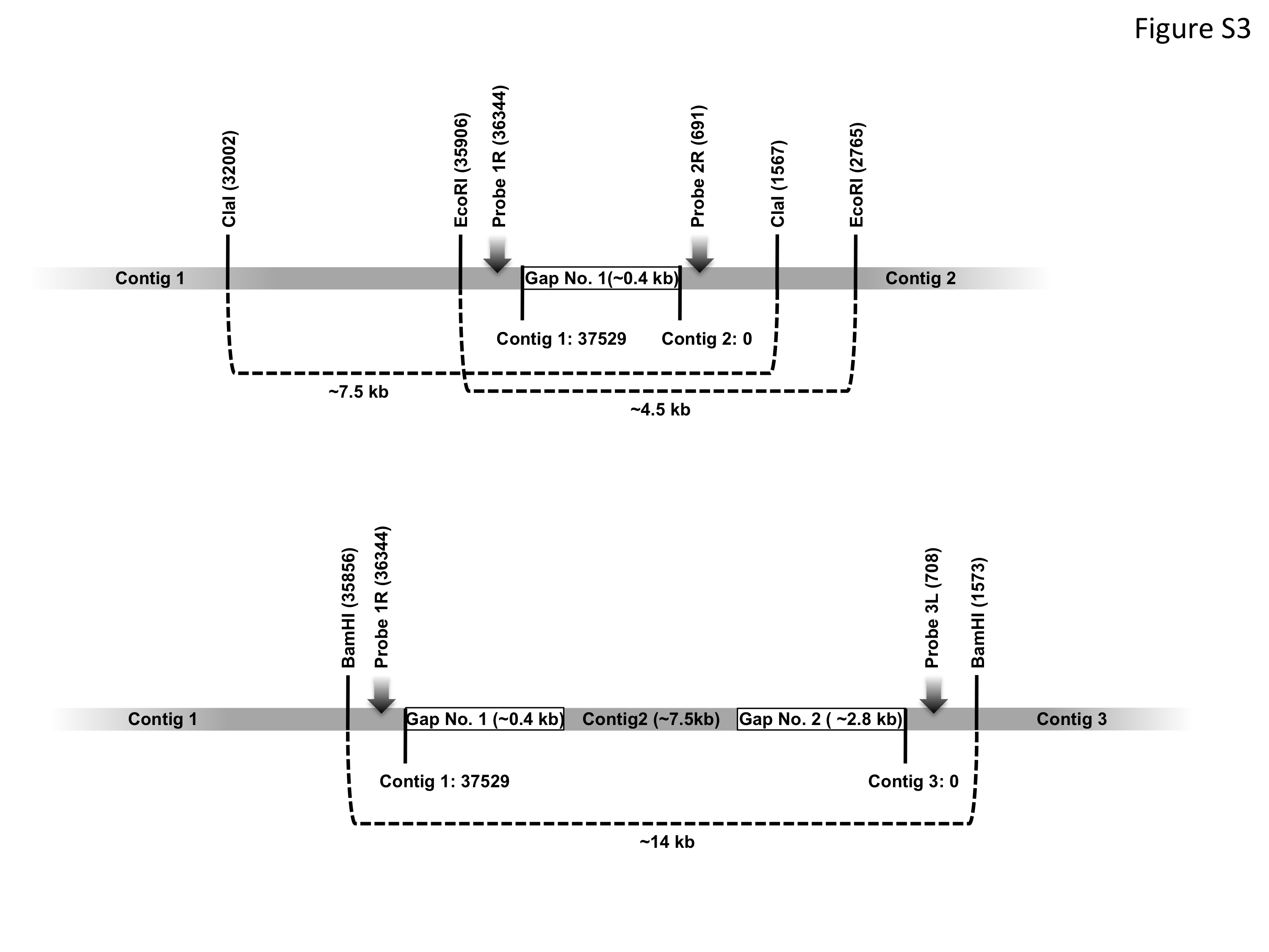

Supplement: Figure S3 — Estimations of the sizes of the gaps within the PR locus assembly based on Southern blotting. The numbers in the parentheses indicate the locations of the restriction enzyme recognition sites within their respective contigs. Block arrows show the locations of the probes used for Southern blotting (see images in Figure S2). The numbers below the dotted lines are the size estimates of the fragments produced from digestions by restriction enzymes. The gap sizes were calculated by subtracting the size of the digestion fragment with the sequences obtained within the fragment interval. (TIF) [file pgen.1002528.s003.tif]

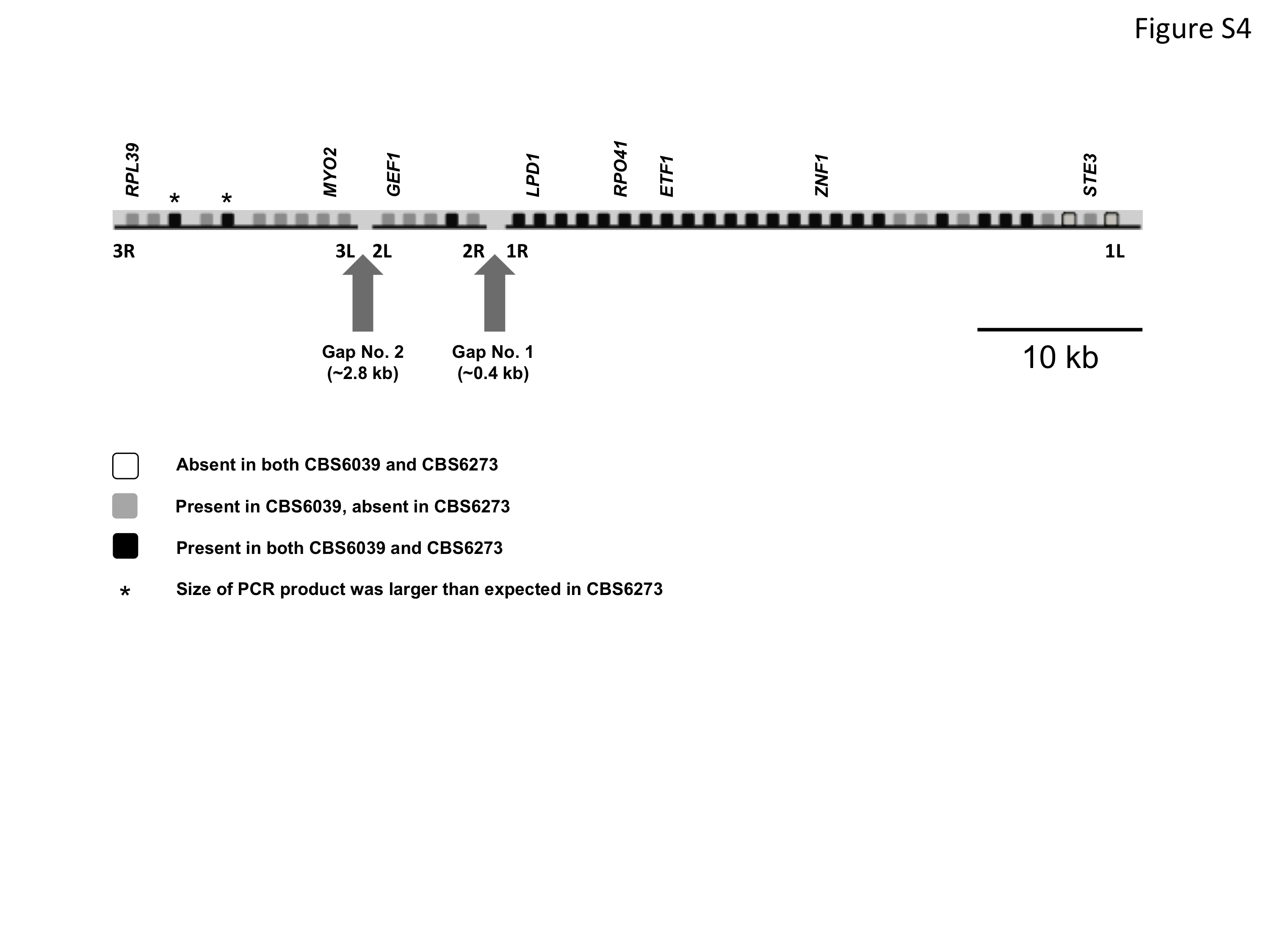

Supplement: Figure S4 — Results of PCR assay indicating similarity and divergence between CBS6039 and CBS6273 within the P/R locus assembly. Primers were designed based on the CBS6039 P/R locus assembly, and were used for PCR reactions using either CBS6039 or CBS6273 genomic DNA as template. Black squares indicate primer pairs that produced PCR products in both strains; gray squares indicate primer pairs that yielded PCR products for CBS6039 but not for CBS6273; white squares indicate primer pairs that yielded no PCR product for either strain. Genes from the CBS6039 P/R locus assembly are labeled at the top. Gaps No.1 and No.2, as well as the contig end labels (1L, 1R, 2L, 2R, 3L, and 3R) correspond to those in Figures S2 and S3. (TIF) [file pgen.1002528.s004.tif]

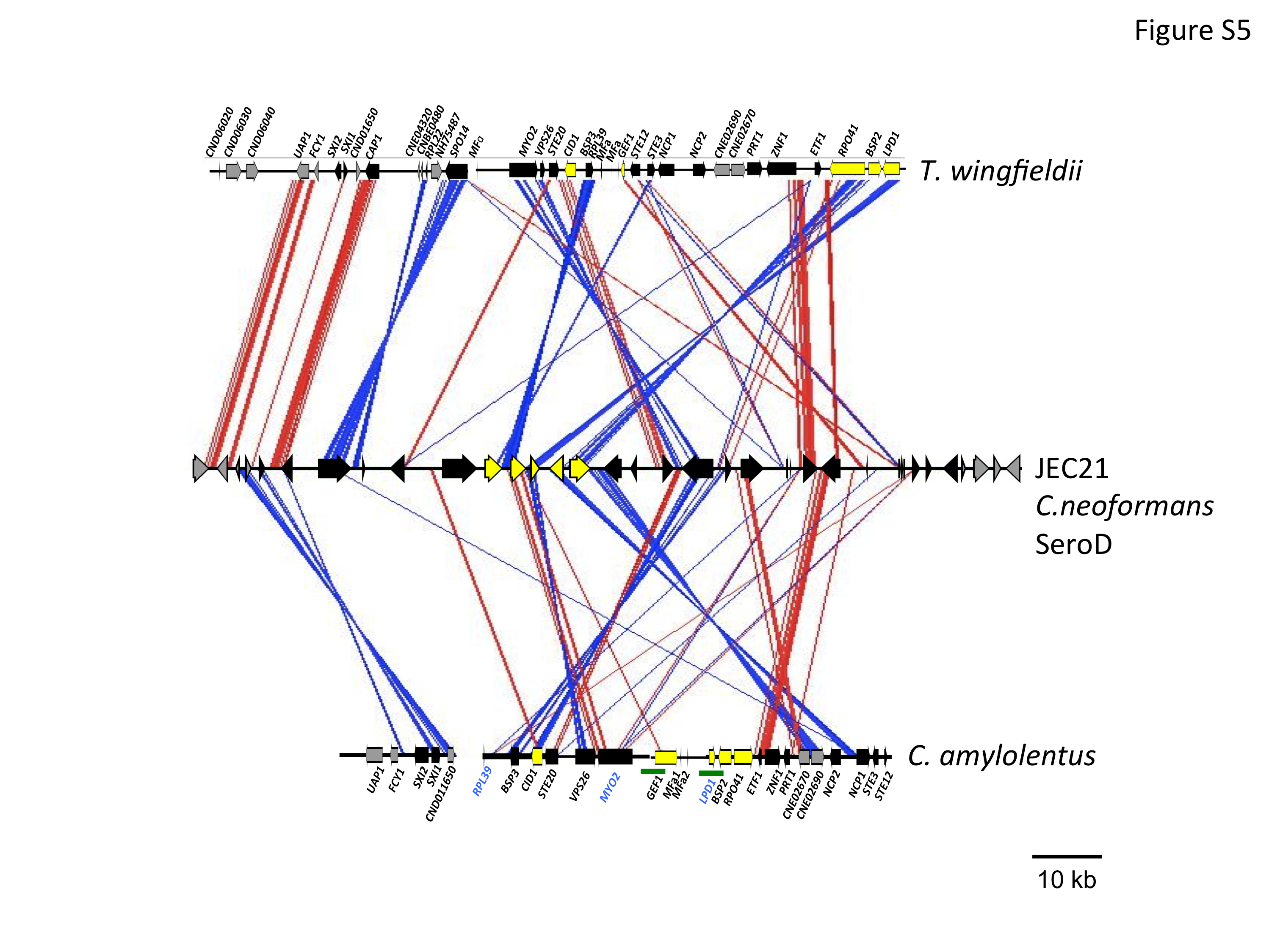

Supplement: Figure S5 — Extensive chromosomal rearrangements are present throughout MAT in the sibling species and C. neoformans strain JEC21. MAT sequences from T. wingfieldii and C. amylolentus were compared to C. neoformans and a synteny analysis was performed. Red denotes conserved gene order while blue indicates inversion events. Green bars under the assembly denote gaps in assembly. (TIF) [file pgen.1002528.s005.tif]

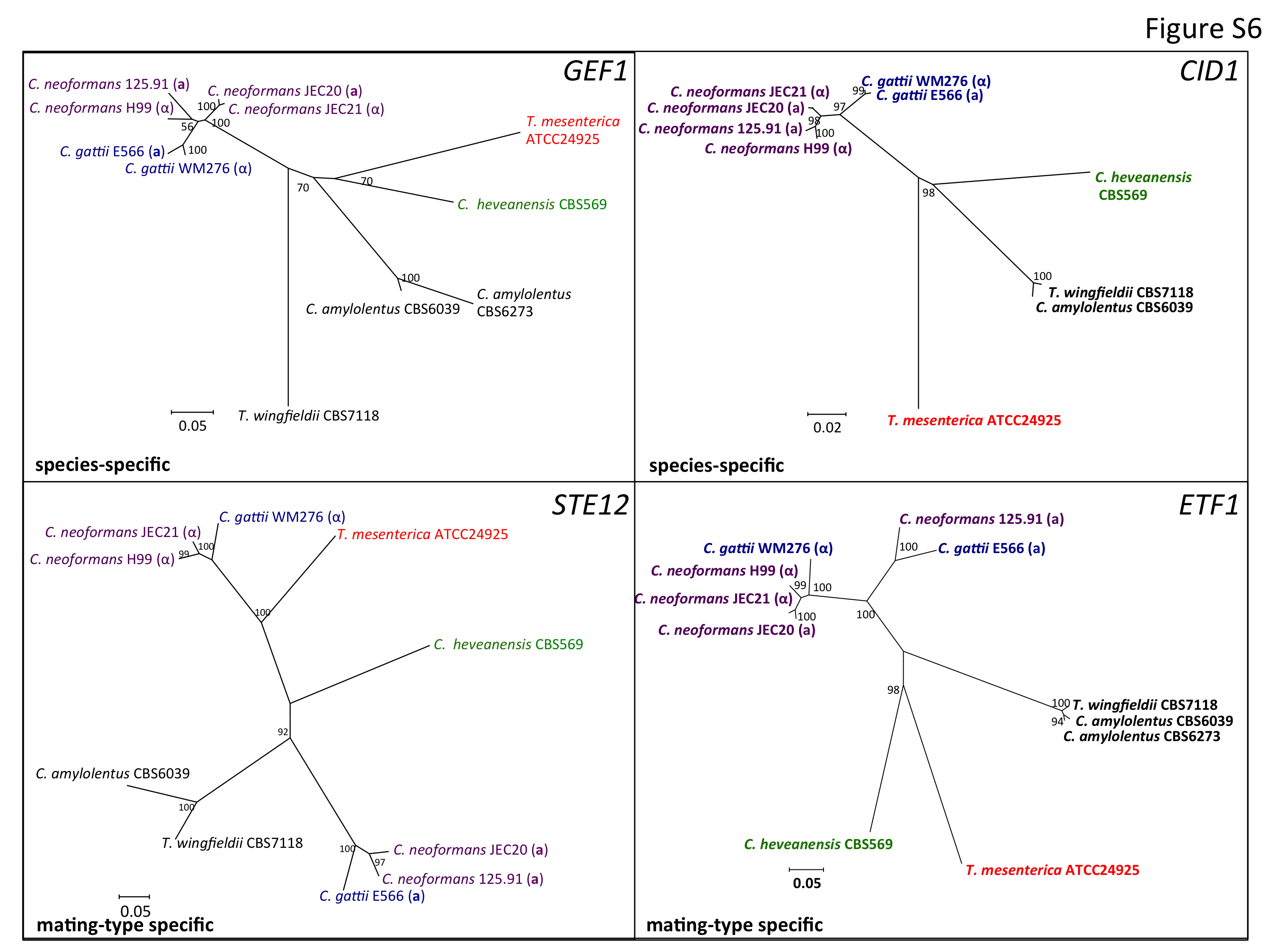

Supplement: Figure S6 — Phylogenetic analysis of additional C. amylolentus genes. The phylogenetic relationship of C. amylolentus and T. wingfieldii to the pathogenic Cryptococcus species and neighboring taxa is highlighted and four representative genes GEF1, CID1, STE12, and ETF1 are shown. GEF1 and CID1 exhibit a species-specific phylogeny in C. neoformans and C. gattii, while STE12 and ETF1 exhibit mating-type specific phylogeny. The trees were constructed using the Neighbor-Joining method implemented in the software MEGA4. Bootstrap values on tree branches were calculated from 500 replicates. (α) indicates strains with the MATα locus, and (a) indicates strains with the MATa locus. (TIF) [file pgen.1002528.s006.tif]

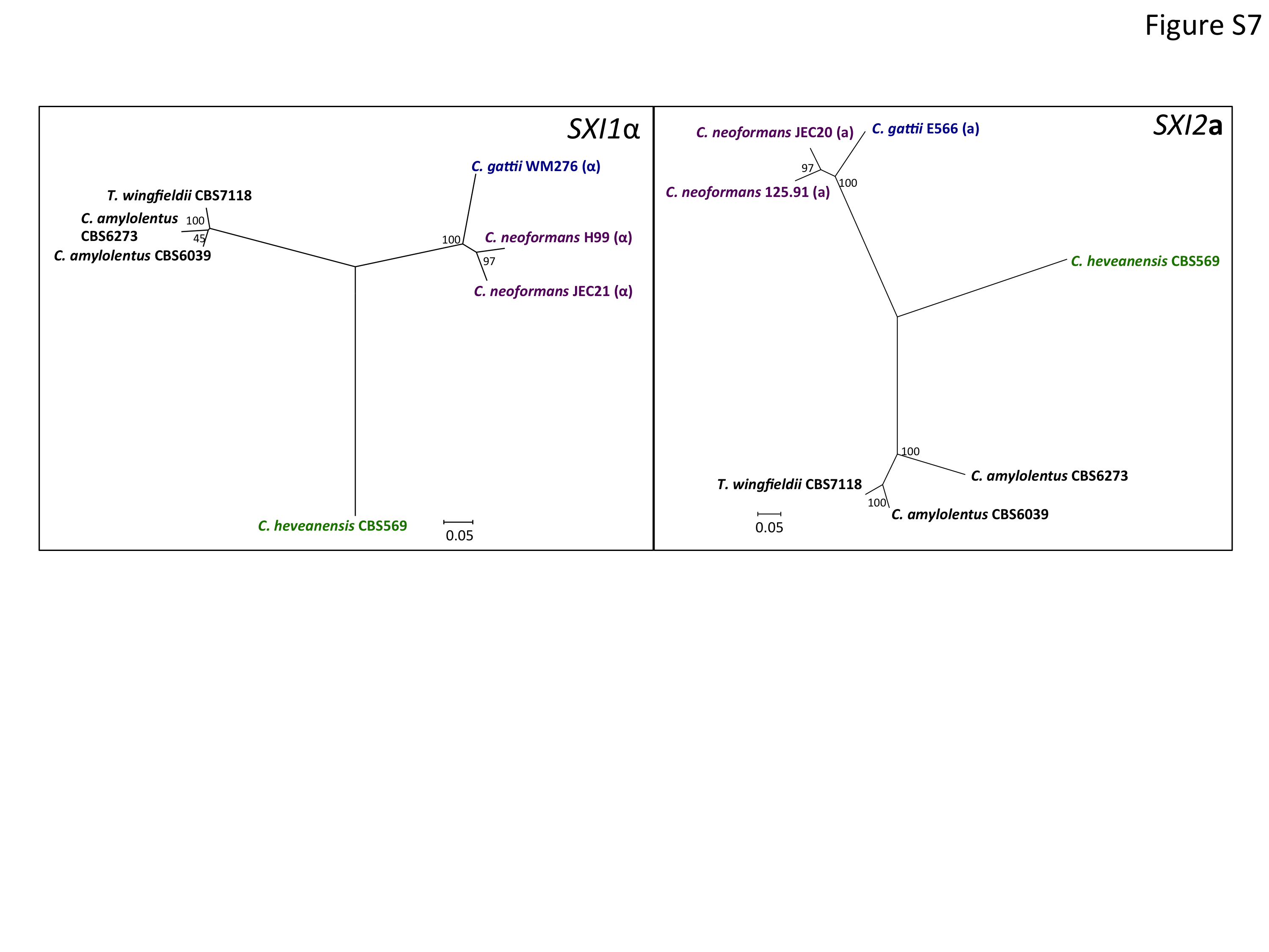

Supplement: Figure S7 — Phylogenetic analysis of the homeodomain transcription factor genes, SXI1 and SXI2 in C. amylolentus. The SXI1 and SXI2 genes were analyzed in C. amylolentus and neighboring taxa and the gene trees were constructed using the Neighbor-Joining method implemented with the software program MEGA4. Bootstrap values on tree branches were calculated from 500 replicates. (α) indicates strains with the MATα locus, and (a) indicates strains with the MATa locus. (TIF) [file pgen.1002528.s007.tif]

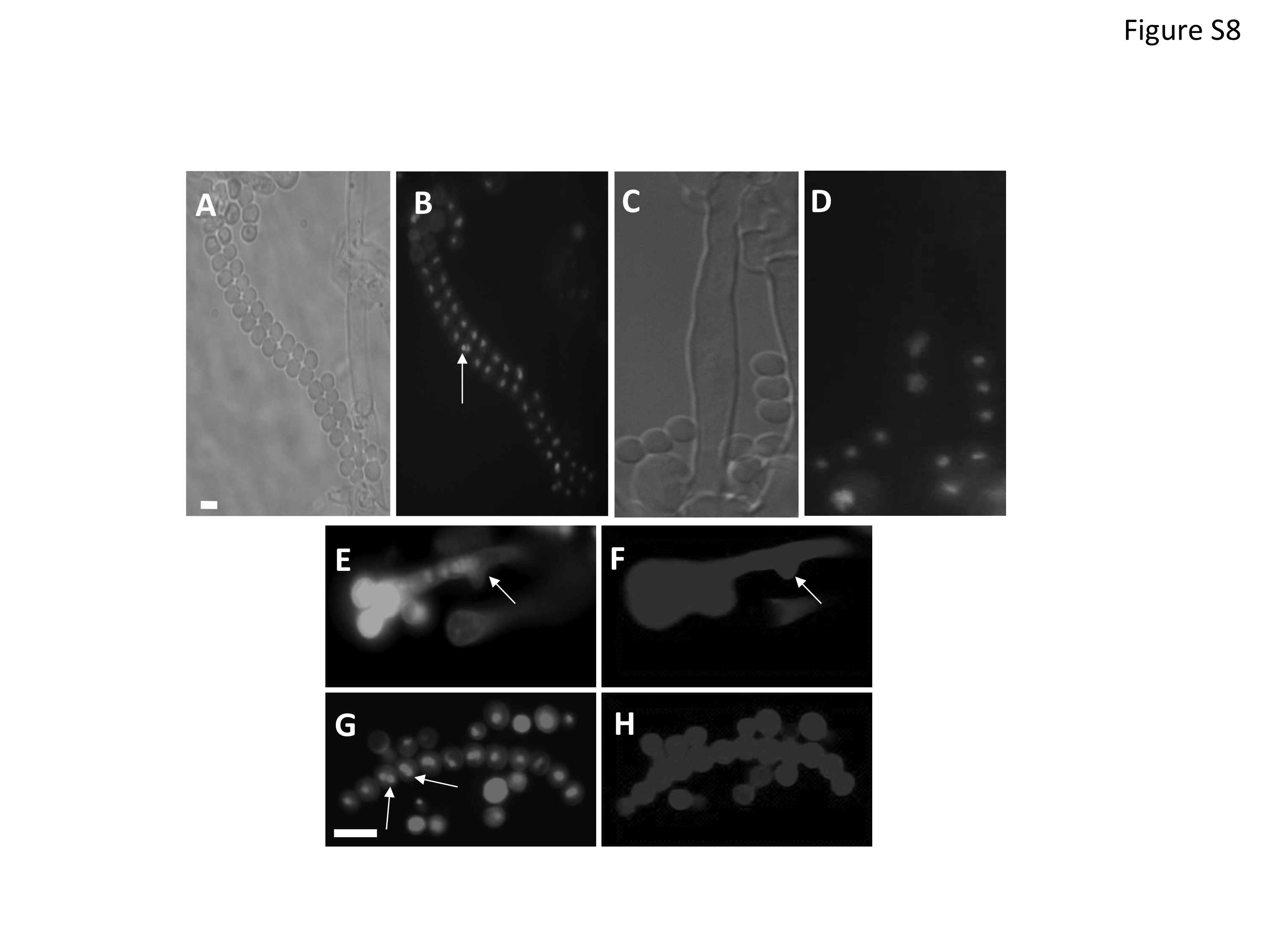

Supplement: Figure S8 — Fluorescence microscopy of C. amylolentus mating structures. (A and B) Staining of basidiospores, scale bars = 2 µm. (A) Differential Interference Contrast (DIC) image, and (B) fluorescence image of basidiospores nuclei stained with Hoechst 33258. (C and D) Staining of a dikaryotic mating filament. (C) DIC image, and (D) fluorescence image of mating filaments, in which nuclei were stained with Hoechst 33258. (E–F) Staining of mating filament and clamp cell. (E) Nuclear content of filament stained with Sytox green and (F) Calcofluor white for cell wall visualization. (G) Nuclear content of basidiospores stained with Sytox green and (H) cell wall with Calcofluor white (scale bar = 5 µm). (TIF) [file pgen.1002528.s008.tif]

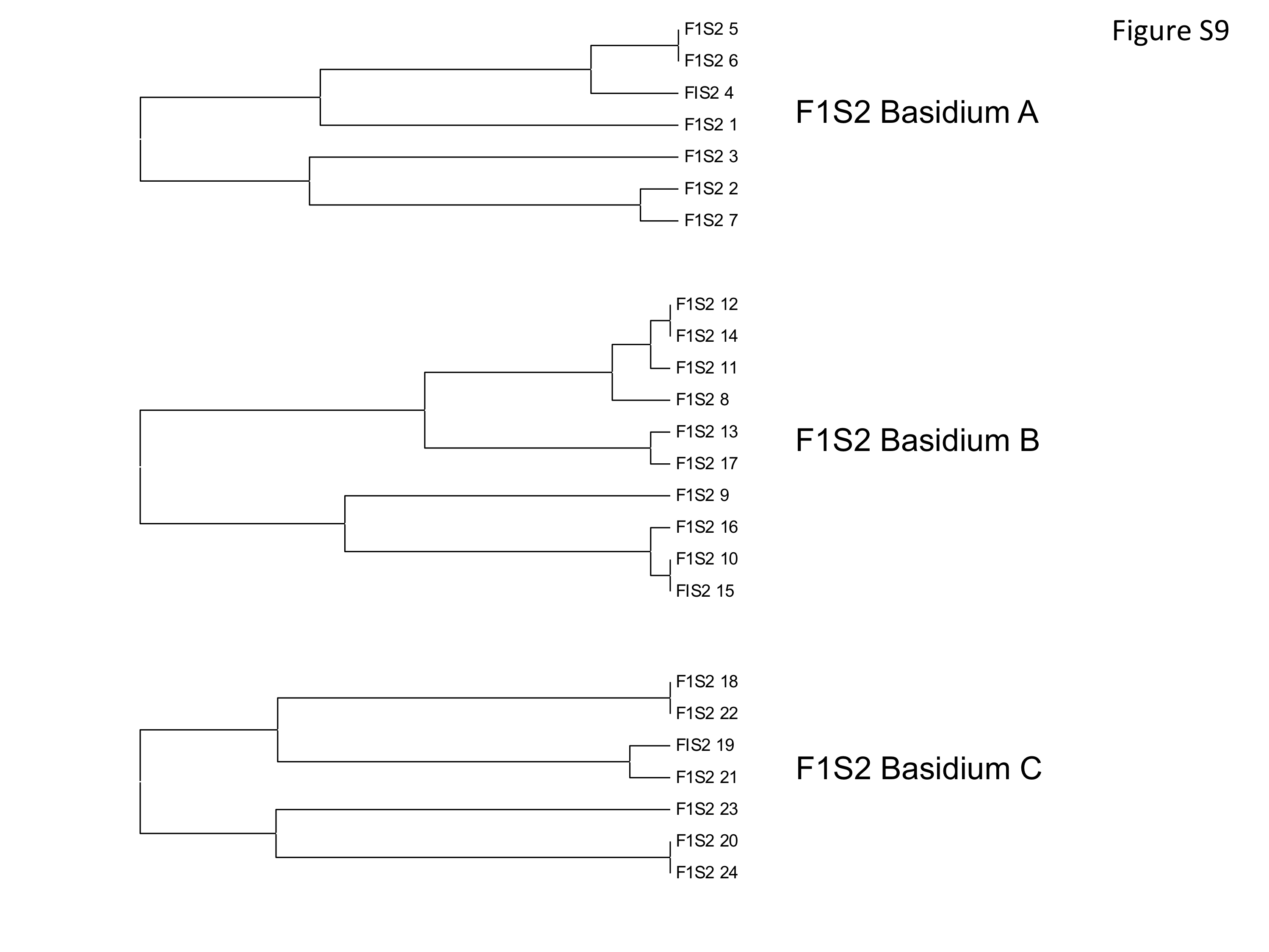

Supplement: Figure S9 — Evidence for one meiotic event in each basidium. Three basidia were analyzed: basidium A includes F1S2 progeny 1 to 7; basidium B includes F1S2 progeny 8 to 17; basidium C includes F1S2 progeny 18 to 24. The genetic distances were calculated using the UPGMA method implemented with the MEGA 5 program. For all three basidia, each harbors four major clusters of consensus progeny genotypes. Some clusters have several progeny that are closely related yet slightly differ from a majority genotype. This is consistent with a scenario in which one meiosis event occurred during sexual reproduction, and some atypical progeny are aneuploid at a few genetic loci from the consensus meiotic genotypes. (TIF) [file pgen.1002528.s009.tif]

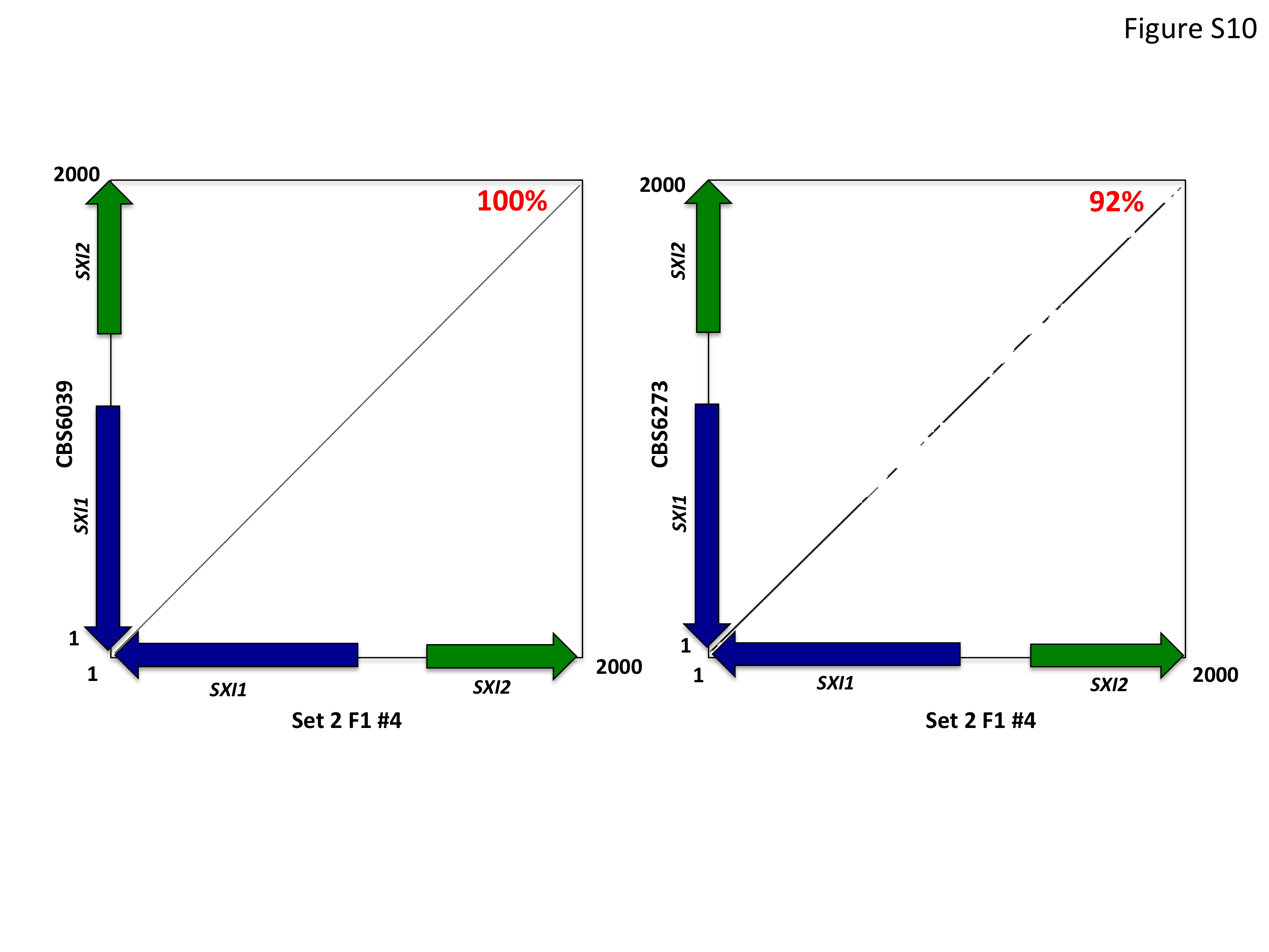

Supplement: Figure S10 — The SXI1 and SXI2 dimorphic region is similar in CBS6039 and F1 set 2 progeny #4. Percent identity plots comparing the ∼2 kb region containing the HD genes in the C. amylolentus parental strain CBS6039 compared to set 2 F1 progeny #4. (TIF) [file pgen.1002528.s010.tif]

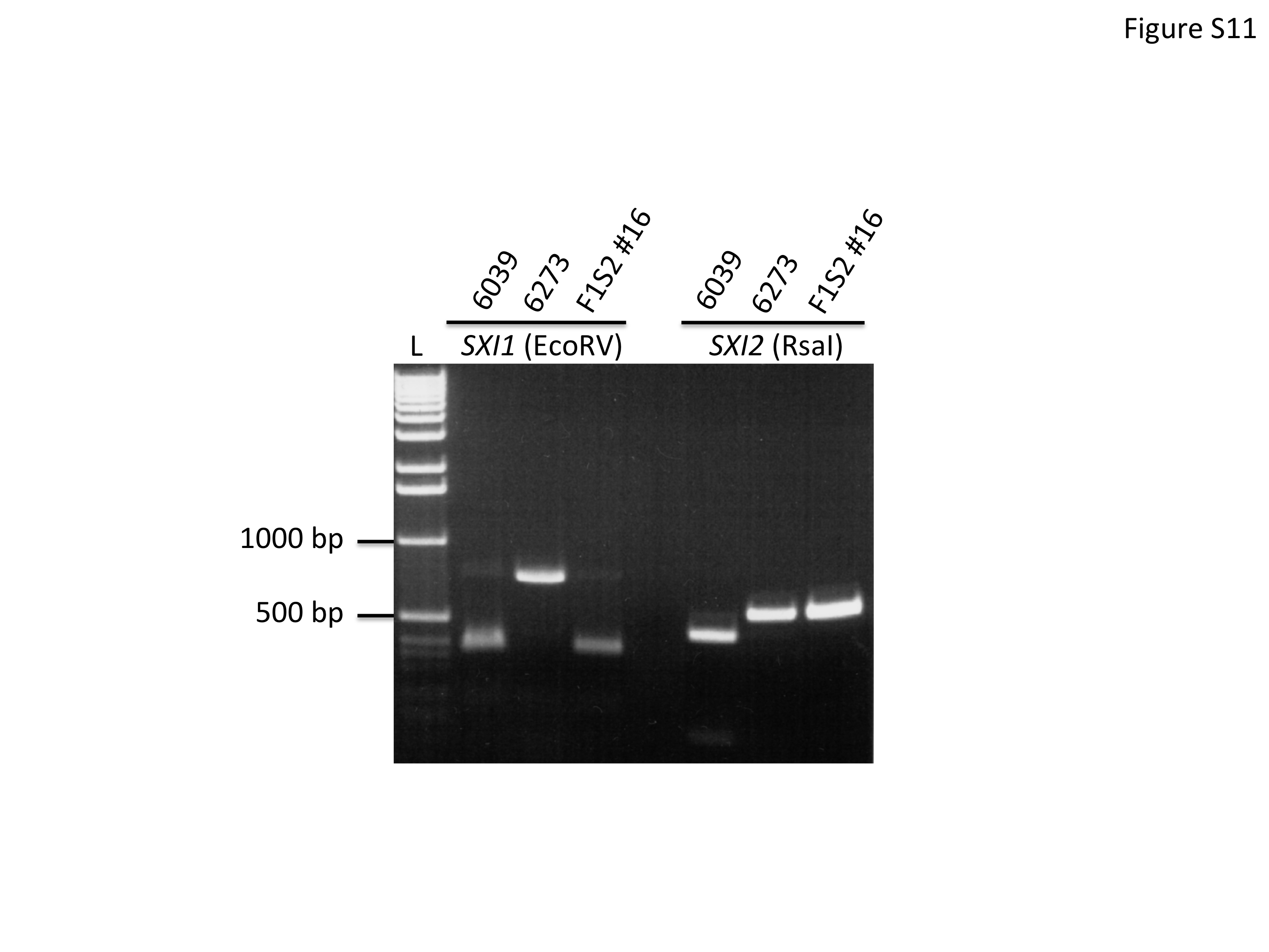

Supplement: Figure S11 — F1 Set 2 progeny #16 is recombinant and types as CBS6039 at SXI1 and as CBS6273 at SXI2. RFLP analysis of the recombinant F1S2 progeny #16. The progeny and both parental strains were digested with EcoRV and RsaI at SXI1 and SXI2, respectively. This analysis reveals a short gene conversion track or very local double crossover event resulted in a novel B MAT locus marker allele. These enzymes only cleave the PCR product in the parental strain CBS6039. L = 1 kb ladder. (TIF) [file pgen.1002528.s011.tif]

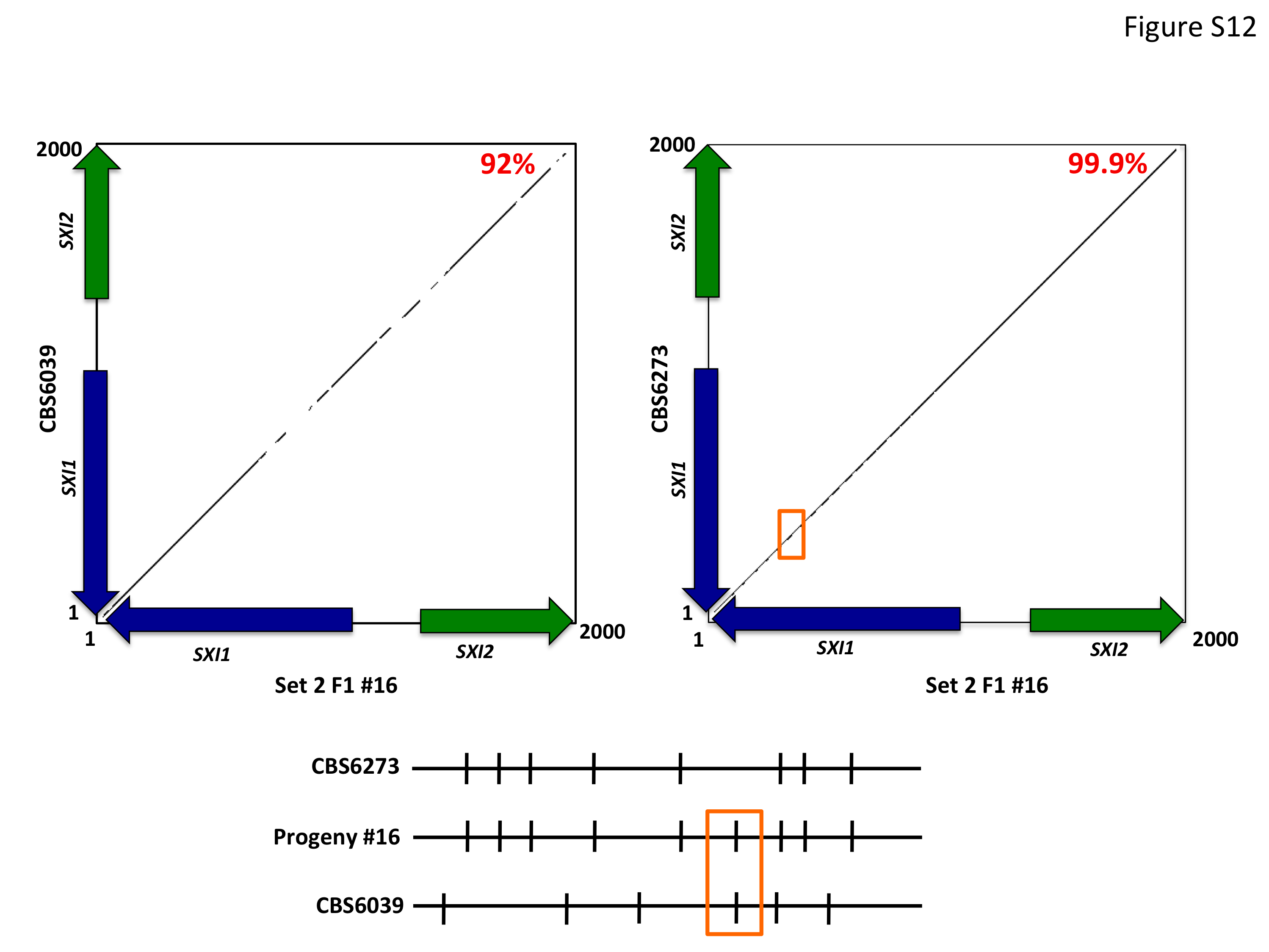

Supplement: Figure S12 — The SXI1-SXI2 dimorphic region differs between CBS6273 and progeny set 2 F1 progeny #16. Percent identity plots comparing the ∼2 kb region containing the HD genes in the C. amylolentus parental strain CBS6273 compared to set 2 F1 progeny #16. An example of the region of crossover or gene conversion in the HD locus of the recombinant progeny #16 is highlighted with an orange box. (TIF) [file pgen.1002528.s012.tif]

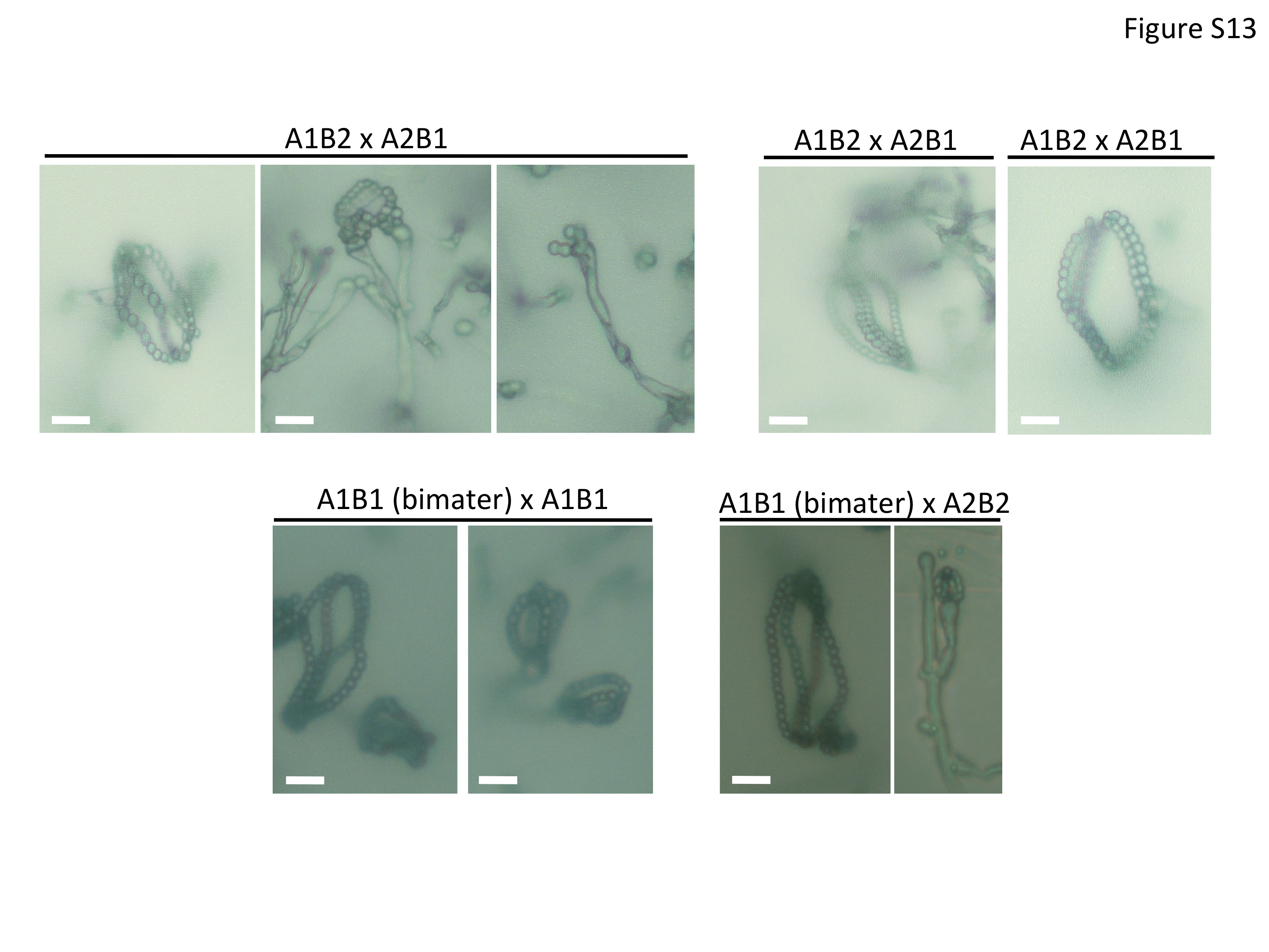

Supplement: Figure S13 — Mating assays between MAT recombinant progeny and the bi-mater set 1 F1 #4 crossed to the parental strains. Light microscopy of mating structures produced in the interfertile progeny (top row, Left panels - F1 set 2 #10×F2 #1 and right panels - F1 Set 2 #16×F2 #1 and F1 Set 2 #16×F2 #5) and backcrosses of progeny #4 to CBS6039 and CBS6273 (bottom row). Scale bar = 10 µm. Mating assays were performed as described in the Materials and Methods. Briefly, the two strains were mixed, and the mixture was spotted onto a V8 (pH = 5) medium plate and incubated in the dark at room temperature for four weeks. (TIF) [file pgen.1002528.s013.tif]
