# Supplementary material for: Discovery of a Modified Tetrapolar Sexual Cycle in Cryptococcus amylolentus and the Evolution of MAT in the Cryptococcus Species Complex
Source: PLoS Genet. 2012 Feb 16;8(2):e1002528. doi: 10.1371/journal.pgen.1002528 (PMC3280970; doi:10.1371/journal.pgen.1002528)
Supplement: Table S1 — Random spore dissection of set 1 F1 progeny and molecular analysis of the nuclear markers. (DOCX) [file pgen.1002528.s014.docx]

Table S1. Random spore dissection of set 1 F1 progeny and molecular analysis of the nuclear markers

| F1 Set 1 | Mating as | B locus ^1^ | |  | A locus ^1^ | | | |
| --- | --- | --- | --- | --- | --- | --- | --- | --- |
|  |  | *SXI2* | *SXI1* |  | *RPL39* | *GEF1* | *ETF1* | *STE3* |
| 1 | sterile | a | a |  | a | a | a | a |
| 2 | A1B1 | a | a |  | a | a | a | a |
| 3 | sterile | a | a |  | a | a | a | a |
| 4 | A1B1 | a | a |  | a | a | a | a |
| 5 | A1B1 | a | a |  | a | a | a | a |
| 6 | sterile | a | a |  | a | a | a | a |
| 7 | sterile | a | a |  | a | a | a | a |
| 8 | sterile | a | a |  | a | a | a | a |
| 9 | sterile | a | a |  | a | a | a | a |
| 10 | sterile | a | a |  | a | a | a | a |
| 11 | sterile | a | a |  | a | a | a | a |
| 12 | sterile | a | a |  | a | a | a | a |
| 13 | sterile | a | a |  | a | a | a | a |
| 14 | sterile | a | a |  | a | a | a | a |
| 15 | A1B1 | a | a |  | a | a | a | a |
| 16 | sterile | a | a |  | a | a | a | a |
| 17 | sterile | b | b |  | a | a | b | a |
| 18 | A2B1 | a | a |  | b | b | b | b |
| 19 | A1B1 | a | a |  | a | a | a | a |
| 20 | sterile | a | a |  | a | a | a | a |
| 21 | sterile | a | a |  | a | a | a | a |
| 22 | sterile | a | a |  | a | a | a | a |
| 23 | sterile | a | a |  | a | a | a | a |
| 24 | sterile | a | a |  | a | a | a | a |
| 25 | sterile | a | a |  | a | a | a | a |
| 26 | sterile | a | a |  | a | a | a | a |
| 27 | sterile | a | a |  | a | b | b | b |
| 28 | sterile | a | a |  | a | b | b | b |

Yellow highlight indicates progeny that are fertile with parental strain;

Blue highlight indicates progeny that are fertile with siblings from F1 set 2 progeny;

^1^: “a” represents allele from A1B1 parent CBS6039; “b” represents allele from A2B2 parent CBS6273; red font indicates recombinant progeny.
