# Supplementary material for: Discovery of a Modified Tetrapolar Sexual Cycle in Cryptococcus amylolentus and the Evolution of MAT in the Cryptococcus Species Complex
Source: PLoS Genet. 2012 Feb 16;8(2):e1002528. doi: 10.1371/journal.pgen.1002528 (PMC3280970; doi:10.1371/journal.pgen.1002528)
Supplement: Table S2 — RAPD analysis of F1 set 1 progeny. (DOCX) [file pgen.1002528.s015.docx]

Table S2. RAPD analysis of F1 set 1 progeny

|  | OPA4_MODI | OPA5_MODI | Pi_Random_5 | Pi_Random_8 | Pi_Random_9 | Pi_Random_15 | Pi_Random_20 | Pi_Random_21 | Pi_Random_24_No.1 | Pi_Random_24_No.2 | JOHE22492 | JOHE22621 | JOHE22631 | JOHE22643 | JOHE22655_No.1 | JOHE22655_No.2 | JOHE22655_No.3 | JOHE22656_No.1 | JOHE22656_No.2 | JOHE22660 |
| --- | --- | --- | --- | --- | --- | --- | --- | --- | --- | --- | --- | --- | --- | --- | --- | --- | --- | --- | --- | --- |
| F1S1_1 | a | a | a | a | a | a | a | a | a | a | a | a | a | a | a | a | a | a | a | a |
| F1S1_2 | a | a | a | a | a | a | a | a | a | a | a | a | a | a | a | a | a | a | a | a |
| F1S1_3 | a | a | a | a | a | a | a | a | a | a | a | a | a | a | a | a | a | a | a | a |
| F1S1_4 | a | a | a | a | a | a | a | a | a | a | a | a | a | a | a | a | a | a | a | a |
| F1S1_5 | a | a | a | a | a | a | a | a | a | a | a | a | a | a | a | a | a | a | a | a |
| F1S1_6 | a | a | a | a | a | a | a | a | a | a | a | a | a | a | a | a | a | a | a | a |
| F1S1_7 | a | a | a | a | a | a | a | a | a | a | a | a | a | a | a | a | a | a | a | a |
| F1S1_8 | a | a | a | a | a | a | a | a | a | a | a | a | a | a | a | a | a | a | a | a |
| F1S1_9 | a | a | a | a | a | a | a | a | a | a | a | a | a | a | a | a | a | a | a | a |
| F1S1_10 | a | a | a | a | a | a | a | a | a | a | a | a | a | a | a | a | a | a | a | a |
| F1S1_11 | a | a | a | a | a | a | a | a | a | a | a | a | a | a | a | a | a | a | a | a |
| F1S1_12 | a | a | a | b | a | a | a | a | a | a | a | a | b | a | a | a | b | a | a | a |
| F1S1_13 | a | a | a | a | a | a | a | a | a | a | a | a | a | a | a | a | a | a | a | a |
| F1S1_14 | a | a | a | a | a | a | a | a | a | a | a | a | a | a | a | a | a | a | a | a |
| F1S1_15 | a | a | a | a | a | a | a | a | a | a | a | a | a | a | a | a | a | a | a | a |
| F1S1_16 | a | a | a | a | a | a | a | a | a | a | a | a | a | a | a | a | a | a | a | a |
| F1S1_17 | a | a | b | a | b | a | b | b | b | a | a | a | a | a | a | a | a | b | a | b |
| F1S1_18 | b | b | b | b | a | b | b | b | b | a | a | a | a | a | a | a | a | b | a | b |
| F1S1_19 | a | a | a | a | a | a | a | a | a | a | a | a | a | a | a | a | a | a | a | a |
| F1S1_20 | a | a | a | a | a | a | a | a | a | a | a | a | a | a | a | a | a | a | a | a |
| F1S1_21 | a | a | a | a | a | a | a | a | a | a | a | a | a | a | a | a | a | a | a | a |
| F1S1_22 | a | a | a | a | a | a | a | a | a | a | a | a | a | a | a | a | a | a | a | a |
| F1S1_23 | a | a | a | a | a | a | a | a | a | a | a | a | a | a | a | a | a | a | a | a |
| F1S1_24 | a | a | a | a | a | a | a | a | a | a | a | a | a | a | a | a | a | a | a | a |
| F1S1_25 | b | b | a | b | a | a | b | b | b | b | b | a | a | b | b | a | b | b | b | a |
| F1S1_26 | a | a | a | a | a | a | a | a | a | a | a | a | a | a | a | a | a | a | a | a |
| F1S1_27 | a | a | b | a | b | a | b | b | b | a | b | a | a | b | b | b | b | a | a | b |
| F1S1_28 | a | a | b | a | b | a | b | b | b | a | b | a | a | b | b | b | b | a | a | b |

Note: “a” represents CBS6039 allele and “b” represents CBS6273 allele
