# Supplementary material for: Discovery of a Modified Tetrapolar Sexual Cycle in Cryptococcus amylolentus and the Evolution of MAT in the Cryptococcus Species Complex
Source: PLoS Genet. 2012 Feb 16;8(2):e1002528. doi: 10.1371/journal.pgen.1002528 (PMC3280970; doi:10.1371/journal.pgen.1002528)
Supplement: Table S3 — List of primers, RAPD markers, and mitochondrial markers used in the study. (DOCX) [file pgen.1002528.s016.docx]

Table S3. List of primers, RAPD markers, and mitochondrial markers used in the study

| **Primer Name** | **Sequences (5' - 3')** | **Note** |
| --- | --- | --- |
| JOHE14679 | CCACCGGCACCGCNGAYCARAARG | FAO1 forward |
| JOHE14683 | GGGAGGGTACTTCTTCTGGGGNCCYTSDAT | FAO1 reverse |
| JOHE15835 | TCCGACGTCGTCATCCAYGTNYTNGA | NOG2 forward |
| JOHE15840 | GGGATCTTGCCGCGGWTVMARTCRTT | NOG2 reverse |
| JOHE15841 | GCCTCAAGACCGCCTGCRTNGARAARMG | LPD1 forward |
| JOHE15845 | GGAGGGGATGGCGGCRTARTTNACRT | LPD1 reverse |
| JOHE15850 | CCGACAAGATGTGCGTCATHACNATHYT | RPO41 forward |
| JOHE15857 | GCACTCGCCGAGCCARTCYWKDAT | RPO41 reverse |
| JOHE18132 | TCTGTGACCGATGCCAGTGTAG | MYO2 forward |
| JOHE18133 | CCCGTGAATGAGGTATGATGAAG | MYO2 reverse |
| JOHE20326 | TCCCGTTTGCTACCATCTTC | RPL39 forward |
| JOHE20327 | TGCGTGAGGATGAAAGAAGA | RPL39 reverse |
| JOHE20330 | CAATGGCGACAAGAACGACATC | LPD1 forward |
| JOHE20331 | GGCGAAACTCAAGTCAATGCG | LPD1 reverse |
| JOHE21460 | AGGTTTGTCTCGGCTGGAGA | SXI2 forward |
| JOHE21461 | ACCAGATTATGGGCAGACTGCT | SXI2 reverse |
| JOHE21462 | TCGGAGATGTCGGAGAAGG | SXI1 forward |
| JOHE21463 | TCGTGAGTTCTTGCTTTGCTG | SXI1 reverse |
| JOHE22392 | AATCGGGCTGAGG | RAPD marker; OPA4 Modified |
| JOHE22393 | AGGGGTCTTGAGG | RAPD marker; OPA5 Modified |
| JOHE22448 | TAAGTCTGCTTGGAG | RAPD marker; Pi Random 5 |
| JOHE22451 | TAAGTCTGCTTCGGAG | RAPD marker; Pi Random 8 |
| JOHE22452 | CCCTCAAAGACTTGG | RAPD marker; Pi Random 9 |
| JOHE22458 | CCTATCCGATCCTGG | RAPD marker; Pi Random 15 |
| JOHE22463 | AACATGGGTCACGCG | RAPD marker; Pi Random 20 |
| JOHE22464 | TTCTGAAACCAGCCC | RAPD marker; Pi Random 21 |
| JOHE22467 | CGTGCAAGGGAGCACC | RAPD marker; Pi Random 24 |
| JOHE22492 | CTCTCAGACGCACGAAG | RAPD marker; SR2 Random 13 |
| JOHE22508 | GGGAGAATTTGATTCAAGTGCAAC | NAD4 Forward (mitochondrial) |
| JOHE22509 | ATGATGTTGCATCTGGCATCATAC | NAD4 Reverse (mitochondrial) |
| JOHE22510 | CTATTGGTGTTACAGGAGCTCAC | NAD5 Forward (mitochondrial) |
| JOHE22511 | GAGCCTTCATACCTGCCTTATTTGC | NAD5 Reverse (mitochondrial) |
| JOHE22621 | GACTCACTCCAGCCATG | RAPD marker; CE Random 01 |
| JOHE22631 | CGGCAGTGTCTTGGCG | RAPD marker; CE Random 11 |
| JOHE22643 | ATCTGGTCTGGGAAGT | RAPD marker; CE Random 23 |
| JOHE22655 | CTTGCGAAGGTTCCGG | RAPD marker; CE Random 35 |
| JOHE22656 | ACTATCCTGGGCCGTC | RAPD marker; CE Random 36 |
| JOHE22660 | GCTCCATACCGGGCAG | RAPD marker; CE Random 40 |
| JOHE23415 | CCCAAGATCACTTCCTCCAA | RPL22 forward |
| JOHE23416 | GTCCTTAGAGGTGGCAACGA | RPL22 reverse |
| JOHE23993 | AACTTCCTGGCCCTTGTCTT | STE3 forward |
| JOHE23994 | TTATACAACCGATCGCTCGAC | STE3 reverse |
| JOHE24001 | TCCACAGACTCACCCCTCTC | ETF1 forward |
| JOHE24002 | TTTTCGCAACCTCTTCAACC | ETF1 reverse |
| JOHE24616 | AAGCGCAGAAGCTGAAAGAC | GEF1 forward |
| JOHE24618 | GCGCTACCAAAATGTCAAC | GEF1 reverse |
| JOHE24787/SS | TGGCAGAGAAGCAAGGATGG | CBS6039_MATcontigs_Primer1F |
| JOHE24788/SS | AGGACATCGGTCATTGGCGA | CBS6039_MATcontigs_Primer1R |
| JOHE24789/SS | TTCTGTGGCTGACGGCTTCA | CBS6039_MATcontigs_Primer2F |
| JOHE24790/SS | ACCAGCGCCATCGTAGCAAT | CBS6039_MATcontigs_Primer2R |
| JOHE24791/SS | TTGGCCGTCCGGTGGTTTTT | CBS6039_MATcontigs_Primer3F |
| JOHE24792/SS | TGCTGTGCGGCTGATCATGT | CBS6039_MATcontigs_Primer3R |
| JOHE24793/SS | GCTCCGGTCGTATTAGCGACA | CBS6039_MATcontigs_Primer4F |
| JOHE24794/SS | TGGTTCACCAAGGGTCCCAT | CBS6039_MATcontigs_Primer4R |
| JOHE24795/SS | ATGGCAGCCCAACGTAGGAAG | CBS6039_MATcontigs_Primer5F |
| JOHE24796/SS | ATACGAACGTCAGGGCGGAT | CBS6039_MATcontigs_Primer5R |
| JOHE24797/SS | AACACCCTTTCCTTCTCGGC | CBS6039_MATcontigs_Primer6F |
| JOHE24798/SS | AGTGACGATGGCGAGGATGA | CBS6039_MATcontigs_Primer6R |
| JOHE24799/SS | TGGCAAGATGCGCACACGTA | CBS6039_MATcontigs_Primer7F |
| JOHE24800/SS | ATCTCTGGCGGATTGTGGCA | CBS6039_MATcontigs_Primer7R |
| JOHE24801/SS | TCTGTGTGGCATACCACGCT | CBS6039_MATcontigs_Primer8F |
| JOHE24802/SS | CTCCCGCGTCGATCAATGAA | CBS6039_MATcontigs_Primer8R |
| JOHE24803/SS | GGAAAAATCCGAGCTGGTCG | CBS6039_MATcontigs_Primer9F |
| JOHE24804/SS | CCTGCCTTTTTGAAACGCCC | CBS6039_MATcontigs_Primer9R |
| JOHE24805/SS | TGGCTCCCTTTGTCGGACATA | CBS6039_MATcontigs_Primer10F |
| JOHE24806/SS | CAGTGTTACGGTTGCCGGAA | CBS6039_MATcontigs_Primer10R |
| JOHE24807/SS | ATGCCCTCCGGATTCCGAGTA | CBS6039_MATcontigs_Primer11F |
| JOHE24808/SS | ACCGAACCTTGTTGCCACAG | CBS6039_MATcontigs_Primer11R |
| JOHE24809/SS | TGGGCGCATTCTTCTAAGGC | CBS6039_MATcontigs_Primer12F |
| JOHE24810/SS | CTTCTGCGGATGCCGATGAT | CBS6039_MATcontigs_Primer12R |
| JOHE24811/SS | GTTTCGCCTTCCACTTGCAC | CBS6039_MATcontigs_Primer13F |
| JOHE24812/SS | GCCGCGTCCAAAGACTTGTT | CBS6039_MATcontigs_Primer13R |
| JOHE24813/SS | GACCTTTCAGGCGAATTGCG | CBS6039_MATcontigs_Primer14F |
| JOHE24814/SS | TGAAGCCTCTGGCATTCTCC | CBS6039_MATcontigs_Primer14R |
| JOHE24815/SS | AAGAGATCAAGGCGGCTGGT | CBS6039_MATcontigs_Primer15F |
| JOHE24816/SS | ACATCCAGGCAGCATCACGA | CBS6039_MATcontigs_Primer15R |
| JOHE24817/SS | TCAAGAAAGCAGGCGCTGAG | CBS6039_MATcontigs_Primer16F |
| JOHE24818/SS | TCTCGAAGGGCAGCATTGGT | CBS6039_MATcontigs_Primer16R |
| JOHE24819/SS | TATTGGCAGGGACAAGGCGA | CBS6039_MATcontigs_Primer17F |
| JOHE24820/SS | AAGAGTGAGGAAGGGCGAGGT | CBS6039_MATcontigs_Primer17R |
| JOHE24821/SS | TCCACTCCAGTCGAAGGGGAA | CBS6039_MATcontigs_Primer18F |
| JOHE24822/SS | GAAGGTGAAATTGGGGCGGT | CBS6039_MATcontigs_Primer18R |
| JOHE24823/SS | GAATGGACTACGATGCCGCA | CBS6039_MATcontigs_Primer19F |
| JOHE24824/SS | CGCTCCCCTTCCGAGATTTT | CBS6039_MATcontigs_Primer19R |
| JOHE24825/SS | GGGAATATGGGGTCTTCGGAG | CBS6039_MATcontigs_Primer20F |
| JOHE24826/SS | CGGTGATTTGCTCGAGTGGA | CBS6039_MATcontigs_Primer20R |
| JOHE24827/SS | TGTTGTGAGTCACGATCCCG | CBS6039_MATcontigs_Primer21F |
| JOHE24828/SS | TGAATCCGAGGATGCGGTGT | CBS6039_MATcontigs_Primer21R |
| JOHE24829/SS | TTGCATCCGCTAGCAGGGTT | CBS6039_MATcontigs_Primer22F |
| JOHE24830/SS | CACTGAGCTGCACTTCGGAA | CBS6039_MATcontigs_Primer22R |
| JOHE24831/SS | GCGGCATGCGTCCAATATGA | CBS6039_MATcontigs_Primer23F |
| JOHE24832/SS | ACTTTTGTCGGAGCGCGAGA | CBS6039_MATcontigs_Primer23R |
| JOHE24833/SS | AGGATAGGCGCGACCTCTGAA | CBS6039_MATcontigs_Primer24F |
| JOHE24834/SS | GTGCGGGGAAAGAAACTTGG | CBS6039_MATcontigs_Primer24R |
| JOHE24835/SS | AGGCCAATTTGTCGGCAGG | CBS6039_MATcontigs_Primer25F |
| JOHE24836/SS | ATGTGGCGGCTTTCGAGACT | CBS6039_MATcontigs_Primer25R |
| JOHE24837/SS | AGGGAAGGTTGAGGGGCAAA | CBS6039_MATcontigs_Primer26F |
| JOHE24838/SS | ATTGGAGGGCGTCATCCGTT | CBS6039_MATcontigs_Primer26R |
| JOHE24839/SS | TGGCTGCTTTGGTACAAGGG | CBS6039_MATcontigs_Primer27F |
| JOHE24840/SS | TCTCGCCTCAACACTGCGAT | CBS6039_MATcontigs_Primer27R |
| JOHE24841/SS | TGTATGACAGGCAAGCCAGC | CBS6039_MATcontigs_Primer28F |
| JOHE24842/SS | AACCTGTCATGCCCACCCAA | CBS6039_MATcontigs_Primer28R |
| JOHE24843/SS | TGTACGCCAGCAGCCTTGA | CBS6039_MATcontigs_Primer29F |
| JOHE24844/SS | TTTGGGTGCTGAAGTCACGG | CBS6039_MATcontigs_Primer29R |
| JOHE24845/SS | CGAGACAACCTCCCAAACGA | CBS6039_MATcontigs_Primer30F |
| JOHE24846/SS | GAGCCTTGGGGTTTGATGGA | CBS6039_MATcontigs_Primer30R |
| JOHE24847/SS | ACCGGCACATCAGTGCCTTA | CBS6039_MATcontigs_Primer31F |
| JOHE24848/SS | TTCGGCCACCATCAAGTGCT | CBS6039_MATcontigs_Primer31R |
| JOHE24849/SS | CCAGATCAAGGGCTCCCAGAA | CBS6039_MATcontigs_Primer32F |
| JOHE24850/SS | CCTTTCTACCCCGTCCGTCAA | CBS6039_MATcontigs_Primer32R |
| JOHE24851/SS | AACAGCAGTGAAGGCGTCCA | CBS6039_MATcontigs_Primer33F |
| JOHE24852/SS | TGGTGGTTGTGGTGTTGGGT | CBS6039_MATcontigs_Primer33R |
| JOHE24853/SS | TGTGGCATTCGGCGACTTGT | CBS6039_MATcontigs_Primer34F |
| JOHE24854/SS | AAAGGAGGAACCACGCCTTG | CBS6039_MATcontigs_Primer34R |
| JOHE24855/SS | CGCATAAGCCATTGCCATCG | CBS6039_MATcontigs_Primer35F |
| JOHE24856/SS | GTCGCGAACGTCTGCAAGAA | CBS6039_MATcontigs_Primer35R |
| JOHE24857/SS | TCCTTGAGTTCCATCCCCAC | CBS6039_MATcontigs_Primer36F |
| JOHE24858/SS | TTCAAGGTCGACATCTGGCG | CBS6039_MATcontigs_Primer36R |
| JOHE24859/SS | CCCATCTTGCCGCATATACC | CBS6039_MATcontigs_Primer37F |
| JOHE24860/SS | CCATCTCCAAAGCTGGTGGA | CBS6039_MATcontigs_Primer37R |
| JOHE24861/SS | GGAACGCATCTGTGACCGAT | CBS6039_MATcontigs_Primer38F |
| JOHE24862/SS | ATGGGCGTCGGATCTGGATT | CBS6039_MATcontigs_Primer38R |
| JOHE24863/SS | TGCCAAGGTAAGGTGCCATTG | CBS6039_MATcontigs_Primer39F |
| JOHE24864/SS | TCCGTGGCTGTGAACACCAT | CBS6039_MATcontigs_Primer39R |
| JOHE24865/SS | TACCCACAGCTCCCTTTCCT | CBS6039_MATcontigs_Primer40F |
| JOHE24866/SS | ACCGCACACTGCTTCCACAT | CBS6039_MATcontigs_Primer40R |
| JOHE24867/SS | TCATTGCTGCGTGGCTCTCT | CBS6039_MATcontigs_Primer41F |
| JOHE24868/SS | CGGAGATCAGTGTTGCCCAA | CBS6039_MATcontigs_Primer41R |
| JOHE24869/SS | TGCCATGTCAGGTGACTGCCT | CBS6039_MATcontigs_Primer42F |
| JOHE24870/SS | CGAGAAGAACAACAGCCCGT | CBS6039_MATcontigs_Primer42R |
| JOHE24871/SS | TTGGACCGGCAGACATACTGG | CBS6039_MATcontigs_Primer43F |
| JOHE24872/SS | TCAGTGCGATGCCTGTTTGG | CBS6039_MATcontigs_Primer43R |
| JOHE24873/SS | ATCAATCACAAGGCACCCGC | CBS6039_MATcontigs_Primer44F |
| JOHE24874/SS | AAGTGCTTGATGAGCGCCGA | CBS6039_MATcontigs_Primer44R |
| GEF1 left forward | GCCCATGTGCTCTTGTGGAA |  |
| GEF1 left reverse | AGCGCAACCGGGTATAAGGA |  |
| LPD1 right forward | TCGGTTTCCACACCAATGGC |  |
| LPD1 right reverse | TCATCGGAGGCGGTGTCATT |  |
| MFA1 right forward | GGTCTCTCGATCTGCTTGCA |  |
| MFA1 right reverse | CCGATAAATGCCAGGTTGCG |  |
| MYO2 left forward | AACACCCACCACCACAACCA |  |
| MYO2 left reverse | TTCAAGAGCGATGACGGGAC |  |
| RPL39 right forward | ATGCACCAGTTAGTGGGCCCT |  |
| RPL39 right reverse | TGAGTCAACAGCGGAAAGGC |  |
| STE12 left forward | GCATGTAGTGAGTGGGATGG |  |
| STE12 left reverse | TTTTCCCGTCCAGTTTCGCC |  |

Note: primers JOHE24787/SS to JOHE24874/SS are used to generate the supplemental Figure S4. Their order of these primer pairs in the Figure S4 is from the right end of the 1L region to the spot besides *RPL39* gene. Additionally, for Figure S4, primer pair JOHE23993/23994 was used to amplify *STE3*, and primer pair JOHE20326/20327 was used to amplify *RPL39*.
