# Supplementary material for: Discovery of a Modified Tetrapolar Sexual Cycle in Cryptococcus amylolentus and the Evolution of MAT in the Cryptococcus Species Complex
Source: PLoS Genet. 2012 Feb 16;8(2):e1002528. doi: 10.1371/journal.pgen.1002528 (PMC3280970; doi:10.1371/journal.pgen.1002528)
Supplement: Table S4 — Filamentous phenotype and mitochondrial DNA (mtDNA) inheritance identified in all 65 progeny. (DOCX) [file pgen.1002528.s017.docx]

Table S4. Filamentous phenotype and mitochondrial DNA (mtDNA) inheritance identified in all 65 progeny

|  | Strains | Filamentous ^1^ | Mating Type ^2^ | *NAD4* ^3^ | *NAD5* ^3^ |
| --- | --- | --- | --- | --- | --- |
| F1Set1 | 1 | ✖ | Sterile | b | b |
|  | 2 | ✖ | A1B1 | b | b |
|  | 3 | ✖ | Sterile | b | b |
|  | 4 | ✖ | A1B1 | b | b |
|  | 5 | ✖ | A1B1 | b | b |
|  | 6 | ✖ | Sterile | b | b |
|  | 7 | ✖ | Sterile | b | b |
|  | 8 | ✖ | Sterile | b | b |
|  | 9 | ✖ | Sterile | b | b |
|  | 10 | ✖ | Sterile | b | b |
|  | 11 | ✖ | Sterile | b | b |
|  | 12 | ✔ | Sterile | b | b |
|  | 13 | ✖ | Sterile | a | a |
|  | 14 | ✖ | Sterile | b | b |
|  | 15 | ✖ | A1B1 | b | b |
|  | 16 | ✖ | Sterile | a | a |
|  | 17 | ✔ | Sterile | b | b |
|  | 18 | ✔ | A2B1 | b | b |
|  | 19 | ✖ | A1B1 | b | b |
|  | 20 | ✖ | Sterile | b | b |
|  | 21 | ✖ | Sterile | b | b |
|  | 22 | ✖ | Sterile | b | b |
|  | 23 | ✖ | Sterile | b | b |
|  | 24 | ✖ | Sterile | b | b |
|  | 25 | ✔ | Sterile | b | b |
|  | 26 | ✖ | Sterile | b | b |
|  | 27 | ✔ | Sterile | b | b |
|  | 28 | ✔ | Sterile | b | b |
| F1Set2 | 1 | ✖ | sterile | b | b |
|  | 2 | ✖ | sterile | b | b |
|  | 3 | ✔ | A1B1 | b | b |
|  | 4 | ✔ | A1B1+A2B2 | b | b |
|  | 5 | ✔ | sterile | b | b |
|  | 6 | ✔ | sterile | b | b |
|  | 7 | ✖ | sterile | b | b |
|  | 8 | ✔ | A1B1 | b | b |
|  | 9 | ✖ | sterile | b | b |
|  | 10 | ✔ | A1B2 | b | b |
|  | 11 | ✔ | A1B1 | b | b |
|  | 12 | ✔ | A1B1 | b | b |
|  | 13 | ✖ | A2B2 | b | b |
|  | 14 | ✔ | A1B1 | b | b |
|  | 15 | ✔ | sterile | b | b |
|  | 16 | ✔ | A1B2 | b | b |
|  | 17 | ✖ | sterile | b | b |
|  | 18 | ✔ | A1B1 | b | b |
|  | 19 | ✖ | sterile | b | b |
|  | 20 | ✔ | A1B1 | b | b |
|  | 21 | ✖ | sterile | b | b |
|  | 22 | ✔ | A1B1 | b | b |
|  | 23 | ✖ | sterile | b | b |
|  | 24 | ✔ | A1B1 | b | b |
|  | 25 | ✔ | sterile | b | b |
|  | 26 | ✔ | sterile | b | b |
|  | 27 | ✔ | sterile | b | b |
|  | 28 | ✔ | sterile | b | b |
|  | 29 | ✔ | sterile | b | b |
|  | 30 | ✔ | sterile | b | b |
|  | 31 | ✔ | sterile | b | b |
| F2 | F2-1 | ✔ | A2B1 | b | b |
|  | F2-2 | ✔ | A2B1 | b | b |
|  | F2-3 | ✔ | A2B2 | b | b |
|  | F2-4 | ✔ | A2B2 | b | b |
|  | F2-5 | ✔ | A2B1 | b | b |
|  | F2-6 | ✔ | A2B2 | b | b |

Blue highlight: the two progeny that inherited mitochondria from CBS6039;

^1^: Filamentous phenotype was assayed on YPD medium;

^2^: Same as those in Tables 1 and S1;

^3^: “a” represents CBS6039 allele and “b” represents CBS6273 allele.
