# Supplementary material for: Discovery of a Modified Tetrapolar Sexual Cycle in Cryptococcus amylolentus and the Evolution of MAT in the Cryptococcus Species Complex
Source: PLoS Genet. 2012 Feb 16;8(2):e1002528. doi: 10.1371/journal.pgen.1002528 (PMC3280970; doi:10.1371/journal.pgen.1002528)
Supplement: Text S1 — Additional discussions on strains, filamentous phenotype, RPL22 duplication, and analyses of HD dimorphic region in key progeny. (DOCX) [file pgen.1002528.s018.docx]

**Supplemental Information**

**CBS6039 and CBS6273 are isolates from the same species**

We observed phenomenon that are unique to mating between CBS6039 and CBS6273, and deviates from observations in a classic tetrapolar mating system, e.g. a high frequency of sterility and unbalanced mating types observed among meiotic progeny. One possible explanation could be that the two isolates actually belong to two diverging species. Indeed, if CBS6039 and CBS6273 were to belong to two diverging species, the possible reproductive incompatibility established between the two species could have given us results similar to those reported in this study. However, there are four reasons that we conclude that this is not the case and that CBS6039 and CBS6273 are isolates of the same species. First, the 16S rDNA sequences of CBS6039 and CBS6273 are identical, suggesting they belong to the same species. Second, the sequences of gene regions from *EF1*α, mitSSU, and *RPB1* are highly similar (with >99% sequence identity) between CBS6039 and CBS6273. In comparison, between *C. neoformans* var. *grubii* and var. *neoformans* (currently recognized as two varieties of one species but possibly two closely related species), the sequence similarities of these three gene fragments are: ~97% (*EF1α*), ~96% (mitSSU), and ~94% (*RPB1*), respectively. Thus, the sequence divergence between CBS6039 and CBS6273 is considerably lower than that observed between closely related varieties of *C. neoformans*, providing further evidence that these two isolates belong to the same species. Third, the spore germination frequency is quite high (70% and 91% for F1S1 and F1S2, respectively) and this is not what is observed when, for example, *S. cerevisiae* is mated with *S. bayanus* that typically results in very low spore germination (<1%). Moreover, the products of spore germination for *C. amylolentus* CBS6039 crossed CBS6273 are often euploid, haploid fertile progeny. If there were a species boundary, typically meiosis would be significantly impaired and post-zygotic boundaries would be imposed by the mismatch repair system aborting attempted meiotic recombination events. Fourth, we have data showing there is no incompatibility between the *MAT* loci from the two strains, i.e. A1B2 and A2B1 progeny generated from the cross between strains CBS6039 and CBS6273 are fully fertile. Taken together, we conclude that both CBS6039 and CBS6273 belong to *C. amylolentus*, and the observed unique characteristics of the mating between these two isolates are thus likely due to the special properties of the sexual reproduction machinery existed in this species.

**Filamentous phenotype**

We examined all the 65 microdissected progeny produced by *C. amylolentus* sexual reproduction for any obvious phenotypic differences. We observed that a subset of the progeny are self-filamentous on YPD medium, and this phenotype does not appear to segregate with a specific mating-type locus allele (see Table S4). Both CBS6039 and CBS6273 appear to be filamentous on YPD but this phenotype is more extensive for CBS6273. By light microscopy, growth of CBS6273 on YPD results in a mixture of hyphae (including pseudohyphae) and yeast-like cells not immediately apparent when CBS6039 is initially grown on YPD solid medium. Fluorescence microscopy of the hyphal cells reveals monokaryotic filaments while the mating filaments are dikaryotic. In the F1S1 progeny, 6 out of 28 progeny are filamentous on YPD and only one of the filamentous isolates is fertile (F1S1 #18) (Tables S1 and S4). Of the 13 fertile F1S2 progeny, 12 are also filamentous on YPD (Tables 1 and S4). In the F2 progeny, all of the progeny are filamentous and all are fertile (Tables 1 and S4). These results suggest there is a strong association between fertility and extensive filamentous growth on YPD.

***RPL22* duplication in *C. amylolentus***

While the *RPL22* gene is present within *MAT* in *C. neoformans* and *C. gattii*, it is missing from the *C. amylolentus* *MAT* assemblies. We confirmed by PCR that the *RPL22* gene is present in the genome of *C. amylolentus.* Southern blotting using *RPL22* gene fragment as probe showed hybridization signals at locations where chromosomes containing HD and P/R loci are located, suggesting this gene has been duplicated and the two copies are located on separate chromosomes. We hypothesize this may be the result of segmental duplication of the *RPL22* gene, followed by chromosomal translocation or transposition. Additionally, the different copy numbers and locations of the gene *RPL22* also conform the point of view that the evolution of *MAT* loci is an ongoing event.

**Dot plot analysis of key progeny**

Progeny microdissection and genotyping using *MAT*-linked markers revealed that sex in *C. amylolentus* produces progeny that are recombinants at the *MAT* loci. The *MAT* reassorted progeny primarily type as A2B1 (14/65) while 3/65 type as A1B2 at the *MAT* markers. Additionally, mating between these *MAT* recombinant progeny is usually successful, and no *MAT* recombinant was able to mate with either parental strain, further confirming that *C. amylolentus* is a heterothallic species with a tetrapolar mating system.

We extended the dot plot analysis of the dimorphic HD genes to two unusual progeny from the F1S2 progeny, #4 (a bi-mater) and #16 (recombinant for *SXI1* and *SXI2*) (Figures S10-S13). Across the *SXI1* and *SXI2* region analyzed, #4 is completely identical to CBS6039; #4 and CBS6039 share 92% similarity with #16 and CBS6273; and #16 shares 99.9% similarity with CBS6273 (Figures S10-S13). So the bi-mater phenotype of F1S2 #4 is not due to nucleotide polymorphisms within the dimorphic HD region. Instead, F1S2 #4 may have acquired the bi-mater phenotype through acquisition of an additional homeodomain gene that is unlinked to the HD locus, or self-compatible mutations in other yet unidentified HD genes. Progeny F1S2 #16 is sterile and recombinant at the HD locus. *SXI1* and *SXI2* are adjacent and the tight linkage of these genes suggests that crossover events are less likely to occur. The dot plot analysis reveals that in *SXI1* one nucleotide that differs between CBS6273 and F1S2 #16 is shared between CBS6039 and F1S2 #16. The remainder of the *SXI1* and *SXI2* genes of CBS6273 and F1S2 #16 are completely identical. We hypothesize that gene conversion occurred from the CBS6039 allele to the CBS6273 allele. Whether this particular gene conversion event is responsible for the sterility of F1S2 #16 needs to be further investigated.
